# Effects of VR interventions on pediatric ADHD: a systematic review, meta-analysis and exploratory machine-learning analysis

**DOI:** 10.3389/fpsyt.2026.1936884

**Published:** 2026-09-30

**Authors:** Tanyue Zhou, Daoyuan Li, Xuwu Chen, Yufei Xu, Dongmei Dai, Feng Zhang

**Affiliations:** 1 School of Rehabilitation and Exercise Health, Gannan Medical University, Ganzhou, Jiangxi, China; 2 Pediatric Neurorehabilitation Department, Ganzhou Women and Children’s Health Care Hospital, Ganzhou, Jiangxi, China

**Keywords:** attention deficit hyperactivity disorder, children and adolescents, machine learning, meta-analysis, virtual reality

## Abstract

**Objective:**

To synthesize the effects of virtual reality (VR) interventions across clinically distinct ADHD-related outcome domains in children and adolescents, compare the pattern of effects across these domains, and examine the robustness, certainty, and potential sources of between-study heterogeneity.

**Methods:**

PubMed, Embase, Web of Science Core Collection, IEEE Xplore, CENTRAL, ScienceDirect, and CNKI were searched for randomized controlled trials and controlled quasi-experimental studies of VR interventions in children and adolescents with ADHD. Outcomes were grouped into four clinical domains: ADHD/behavioral symptoms, executive/cognitive function, social/emotional function, and motor/visual-perceptual function. Random-effects meta-analyses within these domains were treated as the primary efficacy analyses, while an all-domain study-level composite was retained as a secondary descriptive synthesis. Safety, adherence/retention, and cybersickness were summarized at study level. Meta-regression and exploratory machine-learning analyses were used to investigate heterogeneity and were conducted separately from the meta-analytic pooling.

**Results:**

Thirteen studies involving 928 participants were included, of which 11 contributed to the quantitative syntheses. In the primary outcome-domain analyses, pooled Hedges’ g values were 0.49 (95% CI −0.59 to 1.57) for ADHD/behavioral symptoms, 1.15 (95% CI 0.38 to 1.92) for executive/cognitive function, 0.51 (95% CI 0.08 to 0.93) for social/emotional function, and 0.88 (95% CI 0.02 to 1.74) for motor/visual-perceptual function. The dependence-adjusted comparison did not identify differences among domains (p = 0.624), and under Hartung-Knapp inference only the executive/cognitive confidence interval remained above zero. The secondary all-domain synthesis yielded g = 0.98 (95% CI 0.47 to 1.50), with substantial heterogeneity (I² = 91.7%) and a prediction interval spanning the null (−0.69 to 2.65). Exploratory subgroup, meta-regression, and machine-learning analyses did not identify reliable explanations for heterogeneity. Safety and adherence reporting was heterogeneous; no serious intervention-related adverse event was reported among studies reporting safety, but event incidence could not be estimated. GRADE certainty was very low across all four outcome domains.

**Conclusion:**

Domain-specific syntheses identified positive signals for executive/cognitive, social/emotional, and motor/visual-perceptual outcomes, with the clearest support for executive/cognitive function, whereas the ADHD/behavioral estimate remained uncertain. These findings support further evaluation of VR as an adjunctive intervention, although the current evidence does not support domain superiority or consistent effects across settings. The small and heterogeneous evidence base, substantial risk of bias, and very low GRADE certainty limit confidence in the magnitude and reproducibility of the observed effects.

**Systematic review registration:**

https://www.crd.york.ac.uk/PROSPERO/, identifier CRD420261405127.

## 1 Introduction

Attention-deficit/hyperactivity disorder (ADHD) is one of the most common neurodevelopmental disorders in children and adolescents. It is characterized by developmentally inappropriate inattention, hyperactivity, and impulsivity, and is often accompanied by impairments in executive function, emotional regulation, academic adaptation, and social functioning (1). Epidemiological studies suggest that the global prevalence of ADHD among children and adolescents is approximately 5-8%, although estimates vary across regions, diagnostic criteria, and assessment methods (2, 3). Because ADHD symptoms and related functional impairments may have sustained effects on academic performance, interpersonal relationships, and long-term social adaptation, intervention for ADHD is not only a child mental health priority but also closely related to long-term neurodevelopmental and rehabilitation outcomes (4).

Current treatment for ADHD commonly includes pharmacotherapy, behavioral interventions, cognitive training, and parent management training (5, 6). Previous network meta-analyses have shown that stimulant medications, particularly methylphenidate, have relatively well-established short-term efficacy for core ADHD symptoms in children and adolescents (7). However, pharmacological treatment may be limited by adverse effects and variable long-term persistence (8). Non-pharmacological interventions are also used to target ADHD-related cognitive and behavioral functioning, but their effects vary across approaches and outcome measures (9, 10). Therefore, there remains a need to explore adjunctive non-pharmacological interventions with greater contextual relevance, interactivity, and acceptability.

Virtual reality (VR) provides a new technological approach for ADHD assessment and intervention. Compared with traditional paper-and-pencil tasks or two-dimensional computerized training, VR can simulate real-life contexts such as classrooms, games, physical activities, and social interactions, thereby enhancing immersion, immediate feedback, and ecological validity (11–13). For children and adolescents with ADHD, VR environments may increase engagement through multisensory stimulation and may support individualized intervention through adjustable task difficulty, real-time behavioral recording, and contextualized feedback. In recent years, VR has been applied to attention training, executive function training, emotional regulation, motor coordination, visual perception, and social functioning, with preliminary studies suggesting potential benefits for ADHD-related symptoms and functional outcomes (14–17).

Despite the growing number of studies on VR interventions for ADHD, it remains unclear which clinical domains show the most reproducible signals of benefit and whether the observed effects are robust across analytical assumptions and study settings. Existing studies differ substantially in VR device type, degree of immersion, training content, intervention dose, comparator condition, and outcome measurement. Although previous reviews have summarized VR-based assessment and intervention in ADHD, quantitative synthesis of controlled studies, certainty-of-evidence assessment, and systematic evaluation of effect-size heterogeneity remain limited (11, 15, 18). A single cross-domain estimate therefore cannot distinguish a reproducible domain-specific signal from an average shaped by heterogeneous outcomes and study designs.

Accordingly, this systematic review, meta-analysis, meta-regression, and exploratory machine-learning analysis was designed to identify where signals of benefit were most evident and to define their evidential boundaries. Domain-specific syntheses were treated as the primary efficacy analyses, while a study-level all-domain composite provided a secondary descriptive summary. Dependence-aware sensitivity analyses evaluated robustness to multiple outcomes within studies, GRADE assessed certainty, and meta-regression and machine learning examined whether VR technology, comparator type, intervention duration, and other study-level characteristics could explain heterogeneity.

## 2 Methods

### 2.1 Protocol and reporting guideline

The review protocol was recorded in PROSPERO as a non-public training record (CRD420261405127; https://www.crd.york.ac.uk/PROSPERO/view/CRD420261405127). The review was designed, conducted, and reported in accordance with the Preferred Reporting Items for Systematic Reviews and Meta-Analyses 2020 (PRISMA 2020) statement (19, 20). The review question focused on the effects of virtual reality interventions on attention-deficit/hyperactivity disorder (ADHD)-related symptoms and functional outcomes in children and adolescents.

### 2.2 Literature search and eligibility criteria

PubMed, Embase, Web of Science Core Collection, IEEE Xplore, the Cochrane Central Register of Controlled Trials (CENTRAL), ScienceDirect, and China National Knowledge Infrastructure (CNKI) were systematically searched. Searches were conducted from May 20 to May 21, 2026, covering each database from inception to the search date, and were limited to studies published in English or Chinese. The search strategy was developed around three core concepts—VR, ADHD, and children/adolescents—and adapted for each database. Database-specific search strategies are provided in **Supplementary Table S1**.

Studies were eligible if they: (1) included children or adolescents aged ≤18 years with a diagnosis of ADHD or clinically significant ADHD symptoms; (2) used VR, including immersive, semi-immersive, non-immersive, or mixed-reality systems, as the core intervention; (3) included a control condition, such as waitlist/no intervention, usual care, medication, or another active comparator; (4) reported at least one quantitative ADHD-related behavioral, cognitive/executive, social/emotional, motor, or visual-perceptual outcome with sufficient data for effect-size estimation; and (5) used a randomized controlled or controlled quasi-experimental design.

Studies were excluded if they focused on adults with ADHD; did not permit separate extraction of data for children or adolescents with ADHD; did not use VR as the core intervention; primarily included participants with other neurodevelopmental disorders without a distinct ADHD sample; were conference abstracts, editorials, letters, or preprints; or lacked accessible full text or sufficient data for effect-size estimation. For quantitative synthesis, a *post hoc*, significance-independent eligibility rule additionally required an eligible between-group comparison that isolated VR from a non-VR comparator.

### 2.3 Study selection and data extraction

All retrieved records were imported into EndNote X9 for deduplication. Additional duplicate records were identified by checking titles, DOIs, and manual verification. Two reviewers independently screened titles and abstracts, assessed full texts, and extracted data. Disagreements were resolved through discussion or, when necessary, consultation with a third reviewer.

Extracted data included study design, participant characteristics, ADHD diagnostic criteria, VR technology type, intervention protocol, comparator condition, outcome measures and reported results, risk-of-bias information, intervention attendance or completion, withdrawals, adverse events, and cybersickness or simulator-sickness assessments. Safety reporting was classified as assessment using a named structured instrument, narrative reporting, or not reported. Lack of study-level safety reporting was not interpreted as absence of an adverse event.

### 2.4 Risk of bias and certainty of evidence assessment

Risk of bias in randomized studies was assessed using the Cochrane Risk of Bias 2 (RoB 2) tool, covering the randomization process, deviations from intended interventions, missing outcome data, outcome measurement, and selection of the reported result (21). Quasi-experimental studies were assessed using ROBINS-I, covering bias due to confounding, participant selection, intervention classification, deviations from intended interventions, missing data, outcome measurement, and selection of the reported result (22). Assessments focused on the immediate post-treatment outcomes included in this review.

RoB 2 and ROBINS-I assessments were interpreted separately according to their original tool-specific rating systems. Two reviewers independently assessed risk of bias, and disagreements were resolved through discussion and consensus. Detailed judgments and supporting rationales are provided in **Supplementary Table S6**.

The certainty of evidence for each of the four primary outcome domains was assessed using the GRADE framework (23, 24), considering risk of bias, inconsistency, indirectness, imprecision, and publication bias. Certainty was rated as high, moderate, low, or very low. GRADE ratings were assigned only to the four primary outcome domains; the secondary all-domain synthesis and exploratory subgroup analyses were not rated separately.

### 2.5 Quantitative analysis and meta-analysis

When multiple eligible outcomes were reported, a *post hoc* outcome-selection hierarchy was applied manually within each distinct outcome construct or measurement framework. Priority was given to the trial-designated primary outcome, followed by the outcome used for sample-size calculation and then a validated global or domain-total score. When no higher-priority measure was available, eligible non-overlapping outcomes at the same level were retained. Total or derived scores were not included together with their component subscales. Selected outcomes were classified into four clinical domains: ADHD/behavioral symptoms, executive/cognitive function, social/emotional function, and motor/visual-perceptual function. For multi-arm trials, one comparator was selected to avoid repeated use of a shared VR group, with priority given to usual care/no intervention/waitlist, followed by an active non-VR behavioral or psychotherapy comparator and then a pharmacological comparator. Outcome and comparator selection were independent of effect magnitude and statistical significance.

Hedges’ g was used as the standardized effect size, with positive values indicating effects favoring VR. Effect directions were harmonized according to the clinical meaning of each measure. Hedges’ g and its sampling variance were calculated from available post-treatment or change-score means, standard deviations, and group sizes. Reported pairwise Cohen’s d values and eligible two-group interaction or adjusted F statistics were converted to Hedges’ g where necessary. Effects derived from approximate conversions were excluded in a sensitivity analysis. Omnibus statistics from multi-arm comparisons were not converted into pairwise effects. The primary time point was the first assessment conducted at the end of the intervention; later follow-up outcomes were analyzed separately.

When a study contributed multiple selected, non-overlapping effects within the same clinical domain, these were combined into an equal-weight study-domain composite. Because within-study correlations among outcomes were not reported, a common sampling-error correlation of ρ = 0.50 was assumed. The variance of the composite was calculated as:Var( $\bar{\text{g}}$) = m^−^²[Σv_i_ + 2ρΣ_i_<_j_√(v_i_v_j_)]. where m denotes the number of effects and v_i_ their sampling variances. Sensitivity analyses were conducted using ρ = 0, 0.25, 0.75, and 1.00. For the secondary all-domain synthesis, study-domain composites were further combined within each study using equal domain weights and the same covariance approach, yielding one study-level all-domain composite per study.

Random-effects models were fitted using restricted maximum likelihood estimation. The four domain-specific syntheses constituted the primary efficacy analyses, whereas the all-domain synthesis was treated as a secondary descriptive analysis. For each synthesis, Hedges’ g, 95% confidence intervals, Cochran’s Q, I², τ², and a 95% prediction interval, where estimable, were reported (25). Hartung–Knapp inference was used to assess the robustness of inference under the small number of studies (26). Within-study dependence was further examined using multilevel random-effects models and correlated-effects robust variance estimation with small-sample correction (27). Differences among outcome domains were examined using a multilevel moderator model accounting for covariance among domain effects contributed by the same study.

Exploratory moderator analyses examined VR technology type, comparator type, and intervention duration. Technology- and comparator-based analyses used one all-domain composite per study, with moderator tests based on both conventional Wald and Hartung–Knapp inference. Intervention duration was examined using random-effects meta-regression (28); for one study reporting a duration of 6–8 weeks, the midpoint was used in the primary analysis and the study was excluded in a sensitivity analysis. Robustness was further assessed using leave-one-study-out analysis and influence diagnostics. Funnel plots, Egger regression (28), the Begg–Mazumdar rank test, and trim-and-fill were used as small-study-effect diagnostics.

### 2.6 Exploratory machine-learning analysis

The exploratory machine-learning analysis included all 45 signed immediate post-treatment outcome-level effects from the 11 studies contributing to quantitative synthesis. To prevent studies reporting more outcomes from receiving disproportionate influence, each study was assigned a total weight of 1, apportioned among its outcomes in proportion to inverse sampling variance. Given the limited number of independent studies, model complexity was restricted to three candidate specifications: an intercept-only benchmark; a ridge regression model including sample and study-context variables (comparison sample size, mean or midpoint age, medication context, and comparator type); and a broader ridge regression model including all 14 candidate features, additionally incorporating VR type, outcome domain, and effect-size source. These models operated at the study/outcome level rather than the individual-participant level.

Model performance was evaluated using nested grouped leave-one-study-out cross-validation. In each outer fold, one entire study and all of its outcomes were held out for testing. Within the remaining studies, the ridge penalty parameter λ was selected by grouped leave-one-study-out validation from 41 logarithmically spaced values between 10^−4^ and 10^4^, minimizing weighted mean squared error. Candidate models were compared using study-balanced root mean squared error (RMSE), mean absolute error (MAE), and out-of-fold R². RMSE gives greater weight to larger prediction errors, whereas MAE reflects the average absolute prediction error; lower values indicate better predictive performance. Out-of-fold R² compares prediction performance with a mean-effect benchmark, with negative values indicating poorer performance than the benchmark. Model selection was based on lower RMSE and MAE together with higher out-of-fold R².

Machine-learning analyses were conducted only after effect-size calculation and were used to explore study- and outcome-level heterogeneity; they did not generate, modify, or reweight the pooled meta-analytic estimates. All analyses were performed in R version 4.6.0. Meta-analysis, multilevel modeling, and effect-size calculations used the metafor package (version 5.0-1), and correlated-effects robust variance estimation used robumeta (version 2.1).

## 3 Results

### 3.1 Study selection and characteristics

Database searches identified 815 records. After removing 547 duplicates, 268 records were screened by title and abstract. Of these, 243 records were excluded, and 25 full-text articles were sought for retrieval. One full-text article could not be obtained; therefore, 24 full-text articles were assessed for eligibility. After excluding 11 articles, 13 studies were included in the systematic review. Reasons for full-text exclusion included study protocols or registry reports, ineligible study designs, and populations or outcomes that did not match the review question. The study selection process is shown in **Figure 1**.

**Figure 1:**
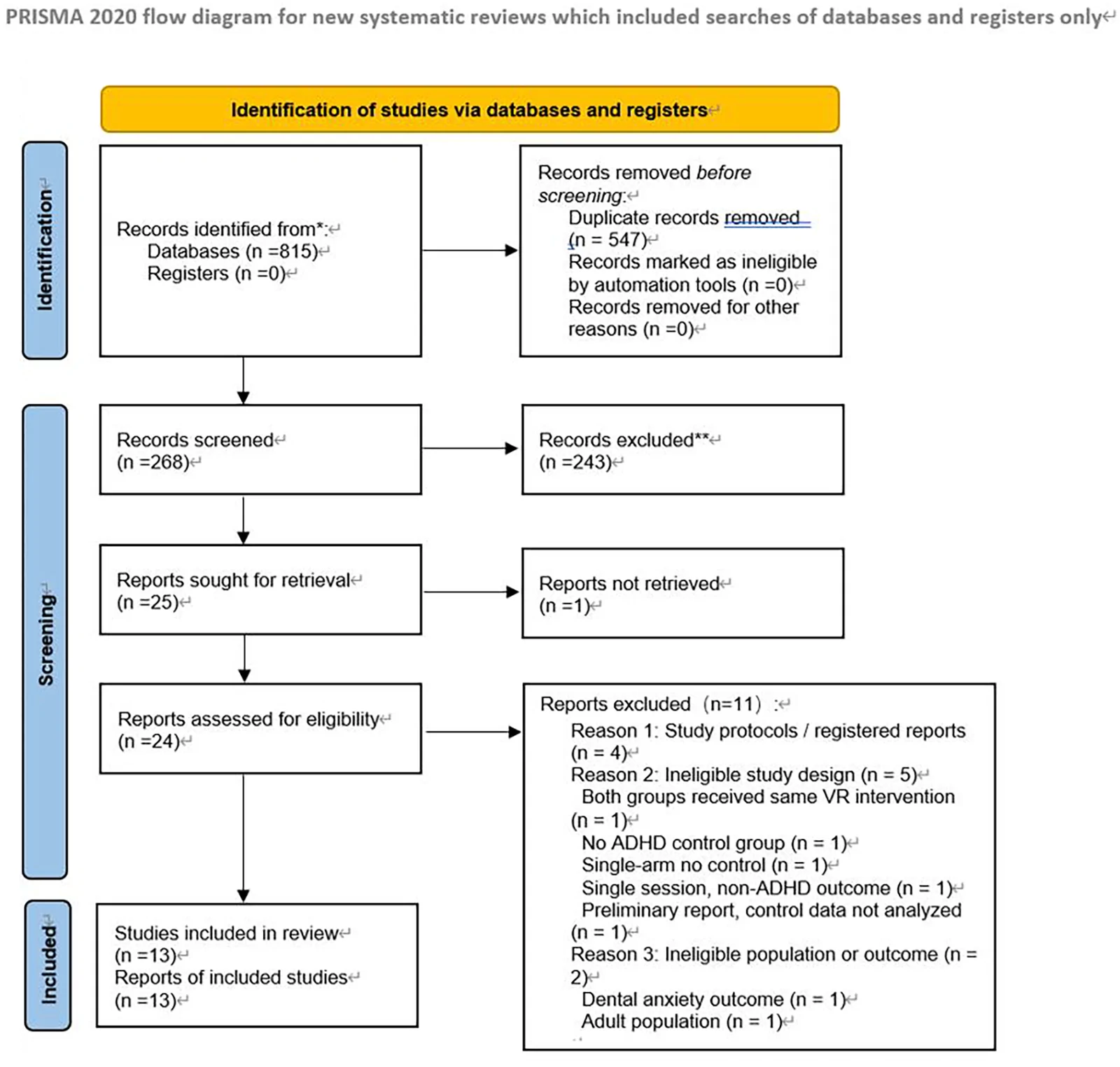
PRISMA flow diagram showing the literature search and study selection process. *Consider, if feasible to do so, reporting the number of records identified from each database of register searched (rather than the total number across all databases/registers). **If automatation tools were used, indicate how many records were excluded by a human and how many were excluded by automation tools.

The 13 included studies comprised 928 children and adolescents with ADHD. Eleven studies were eligible for quantitative synthesis and enrolled 653 participants in total. The selected VR-control comparisons or reported analysis sets used for effect-size estimation included 584 participants; the difference reflects non-selected arms in multi-arm studies and study-specific analyzed-sample counts. Barkin et al. compared therapist-guided with self-directed use of the same game, so the between-group contrast estimated the effect of therapist guidance rather than VR exposure. Fang et al. compared reward-enhanced VR with conventional VR without reward feedback, so the contrast estimated the effect of reward feedback rather than VR exposure. Because neither comparison estimated VR relative to a non-VR comparator, both studies were retained in the qualitative synthesis and risk-of-bias assessment but excluded from the quantitative synthesis. Study and intervention characteristics are summarized in **Table 1**.

**Table 1:** Study, intervention, and quantitative-synthesis characteristics of included studies.

Part A. Study and participant characteristics							
Study	Country/region	Design	Participants (N; age; male %)	Diagnosis			Tool-specific overall RoB
Cai et al., 2025 (56)	China	Quasi-RCT	98; 8.61 ± 1.40 y; 55.1% male	DSM-5			RoB 2: High
Ozkan Yilmaz et al., 2026 (57)	Turkey	RCT	16; 10; range 8–12 y; 81.3% male	DSM-5			RoB 2: High
Tabrizi et al., 2020 (58)	Iran	Quasi-experimental	48; 9.58 ± 1.80 y; 66.7% male	DSM-5			ROBINS-I: Moderate
Martin-Moratinos et al., 2025 (59)	Spain	RCT	76; 12.68 ± 2.75 y; male NR	DSM-5			RoB 2: High
Perez-Rodriguez et al., 2026 (60)	Spain	RCT	80; Range 9–17 y; male NR	DSM-5			RoB 2: Low
Wong et al., 2024 (38)	China (Hong Kong)	RCT	90; 8.53 ± 1.68 y; 66.7% male	DSM-5			RoB 2: Some concerns
Sahin et al., 2026 (61)	Turkey	RCT	60; Range 7–12 y; male NR	DSM-5			RoB 2: Some concerns
Kim et al., 2020 (62)	Korea	RCT	40; Range 7–10 y; male NR	DSM-5			RoB 2: High
Köse et al., 2023 (63)	Turkey	RCT	176; Range 6–12 y; 75.6% male	DSM-5			RoB 2: Some concerns
Guler et al., 2026 (64)	Turkey	Single-blind RCT	70; 9 y; 60.0% male	DSM-5			RoB 2: High
Fang et al., 2025 (39)	China	Quasi-experimental	99; Range 5–6 y; male NR	NR			ROBINS-I: Serious
Bioulac et al., 2018 (65)	France	RCT	51; 9.30 ± 1.40 y; 78.4% male	DSM-IV			RoB 2: High
Kim et al., 2024 (66)	Korea	RCT	24; Range 6–12 y; 42.0% male	DSM-5			RoB 2: High

Part B. Intervention and quantitative-synthesis characteristics							
Study	VR technology	Comparator	Dose		Eligible immediate post-treatment effects retained, n	Quantitative synthesis	
Cai et al., 2025 (56)	Immersive HMD	Active comparator	480 min		2	Yes	
Ozkan Yilmaz et al., 2026 (57)	Immersive HMD	Medication/usual care	240 min		5	Yes	
Tabrizi et al., 2020 (58)	Non-immersive desktop VR	Waitlist/no intervention	10 sessions		1	Yes	
Martin-Moratinos et al., 2025 (59)	Semi-immersive	Medication/usual care	600 min		1	Yes	
Perez-Rodriguez et al., 2026 (60)	Immersive HMD	Medication/usual care	12 sessions		6	Yes	
Wong et al., 2024 (38)	Immersive HMD	Waitlist/no intervention	240 min		6	Yes	
Sahin et al., 2026 (61)	Immersive HMD	Active comparator	720 min		2	Yes	
Kim et al., 2020 (62)	Mixed reality	Waitlist/no intervention	450 min		5	Yes	
Köse et al., 2023 (63)	Tablet game-based intervention	Self-directed use of same game	720 min		—	No (a)	
Guler et al., 2026 (64)	Semi-immersive	Waitlist/no intervention	16 sessions		11	Yes	
Fang et al., 2025 (39)	Reward-enhanced VR	VR without reward feedback	700 min		—	No (b)	
Bioulac et al., 2018 (65)	Immersive HMD	Active comparator	12–16 sessions		5	Yes	
Kim et al., 2024 (66)	Immersive HMD	Active comparator	120 min		1	Yes	

“Retained effects, n” denotes the number of eligible immediate post-treatment between-group effect estimates used to construct study-domain composites. RoB 2 and ROBINS-I judgments are reported using their original tool-specific categories. NR, not reported; RCT, randomized controlled trial; HMD, head-mounted display; VR, virtual reality.

(a) Excluded from quantitative synthesis because the comparison was between therapist-guided and self-directed use of the same game and therefore did not isolate VR exposure relative to a non-VR comparator.

(b) Excluded from quantitative synthesis because the comparison was between reward-enhanced VR and conventional VR without reward feedback and therefore did not isolate VR exposure relative to a non-VR comparator.

Safety and adherence reporting varied across studies (**Supplementary Table S5**). Four studies used named structured instruments or side-effect scales, six reported harms narratively, and three did not report study-level safety findings. Among studies reporting safety outcomes, no serious intervention-related adverse events were reported, and the reported symptoms were generally mild or transient. Event incidence was not pooled because assessment methods and denominators differed across studies. Adherence was reported using heterogeneous definitions, including attendance, completion, engagement, and attrition, and medication adherence was not systematically reported.

### 3.2 Primary outcome-domain and secondary all-domain syntheses

The 45 eligible immediate post-treatment effects yielded 17 study-domain composites across four clinical domains at the primary working correlation of ρ = 0.50. In conventional REML models, ADHD/behavioral symptoms included four studies (g = 0.49, 95% CI −0.59 to 1.57; p = 0.374; I² = 93.5%), executive/cognitive function included seven studies (g = 1.15, 95% CI 0.38 to 1.92; p = 0.003; I² = 93.5%), social/emotional function included four studies (g = 0.51, 95% CI 0.08 to 0.93; p = 0.019; I² = 60.0%), and motor/visual-perceptual function included two studies (g = 0.88, 95% CI 0.02 to 1.74; p = 0.045; I² = 82.9%). The dependence-adjusted comparison showed no statistically significant difference among the four outcome domains (F(3,13) = 0.60, p = 0.624). Under Hartung–Knapp inference, only the executive/cognitive estimate retained a 95% confidence interval above zero, whereas the other domain estimates were imprecise (**Figure 2A**; **Table 2**; **Supplementary Tables S4A, B**).

**Figure 2:**
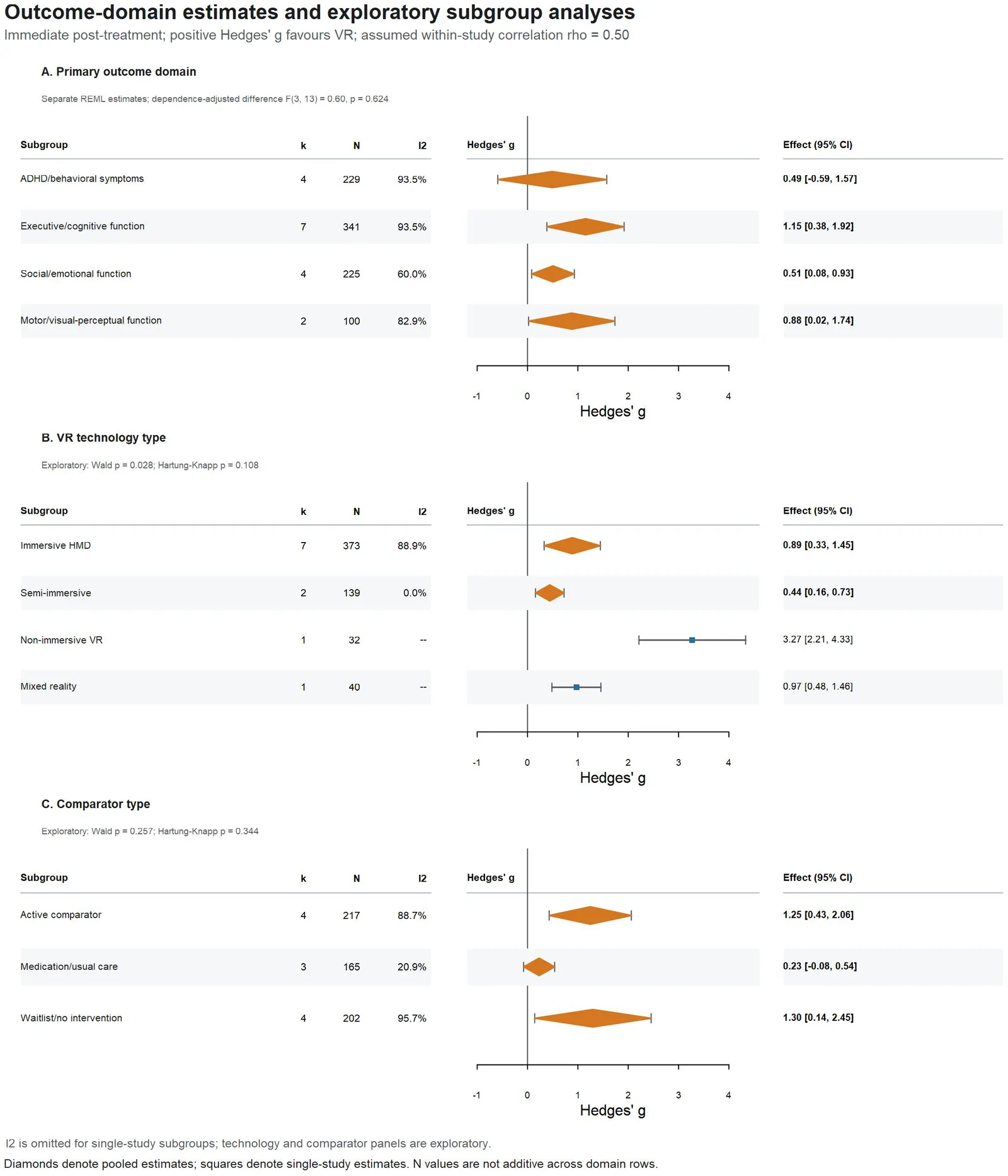
Primary outcome-domain estimates and exploratory subgroup analyses. **(A)** Primary outcome-domain estimates. **(B)** Exploratory subgroup analysis by VR technology type. **(C)** Exploratory subgroup analysis by comparator type. Positive Hedges’ g values favor VR. k, number of studies; N, number of participants; I^2^, heterogeneity.

**Table 2:** Primary outcome-domain, secondary all-domain, and exploratory subgroup analyses.

Analysis	k	N	Hedges’ g (95% CI)	p value	I² (%)	Notes
Primary outcome-domain analyses						Dependence-adjusted F(3,13) = 0.60; p = 0.624
ADHD/behavioral symptoms	4	229	0.49 (-0.59 to 1.57)	0.374	93.5	
Executive/cognitive function	7	341	1.15 (0.38 to 1.92)	0.003	93.5	
Social/emotional function	4	225	0.51 (0.08 to 0.93)	0.019	60.0	
Motor/visual-perceptual function	2	100	0.88 (0.02 to 1.74)	0.045	82.9	
Secondary all-domain synthesis						Descriptive synthesis; 95% prediction interval -0.69 to 2.65
Random-effects summary	11	584	0.98 (0.47 to 1.50)	<0.001	91.7	
Exploratory subgroup analysis: VR technology						Wald p = 0.028; Hartung-Knapp p = 0.108
Immersive HMD	7	373	0.89 (0.33 to 1.45)	0.002	88.9	
Semi-immersive	2	139	0.44 (0.16 to 0.73)	0.002	0.0	
Non-immersive desktop VR	1	32	3.27 (2.21 to 4.33)	<0.001		
Mixed reality	1	40	0.97 (0.48 to 1.46)	<0.001		
Exploratory subgroup analysis: comparator						Wald p = 0.257; Hartung-Knapp p = 0.344
Active comparator	4	217	1.25 (0.43 to 2.06)	0.003	88.7	
Medication/usual care	3	165	0.23 (-0.08 to 0.54)	0.142	20.9	
Waitlist/no intervention	4	202	1.30 (0.14 to 2.45)	0.028	95.7	

Positive Hedges’ g values favor VR. k, number of studies; N, participants in selected comparisons or reported analysis sets; CI, confidence interval; HMD, head-mounted display. N should not be summed across outcome-domain rows because some studies contributed to more than one domain. Outcome-domain analyses are primary, the all-domain synthesis is secondary and descriptive, and technology and comparator analyses are exploratory.

As a secondary descriptive analysis, the 17 study-domain composites were further combined to yield one all-domain effect for each of the 11 quantitative studies. The random-effects estimate was g = 0.98 (95% CI 0.47 to 1.50; p < 0.001), with substantial heterogeneity (I² = 91.7%, τ² = 0.658, Q(10) = 83.13, p < 0.001) and a 95% prediction interval of −0.69 to 2.65. Hartung–Knapp inference yielded g = 0.98 (95% CI 0.37 to 1.60; p = 0.005), while multilevel and robust-variance sensitivity models yielded g = 0.86 (95% CI 0.47 to 1.25) and g = 1.05 (95% CI 0.47 to 1.62), respectively. Varying ρ from 0 to 1 changed the estimate from 0.97 to 1.00. The average estimate was therefore similar across dependence assumptions, although the prediction interval crossed zero (**Figure 3**; **Table 2**; **Supplementary Tables S4A, B**).

**Figure 3:**
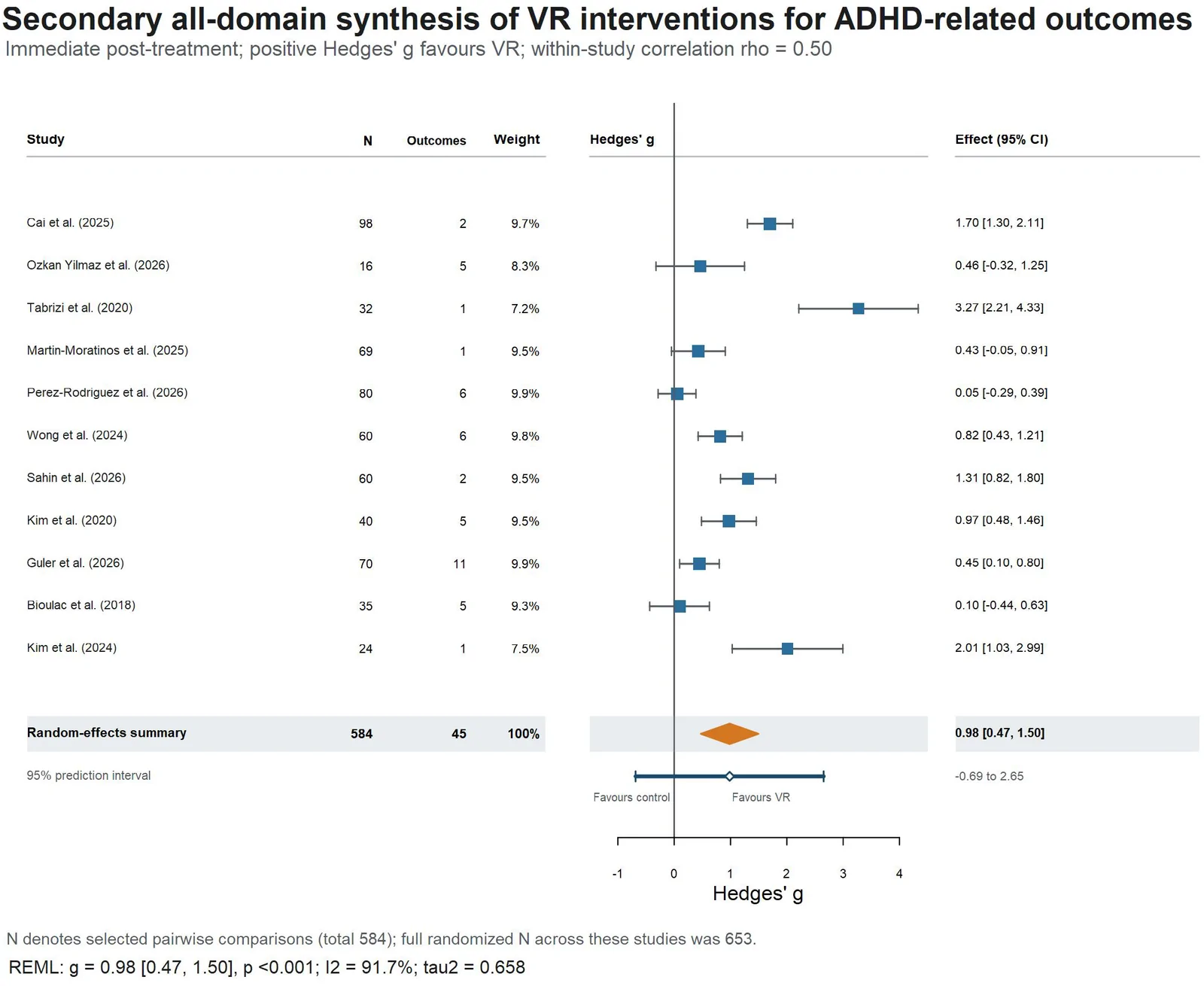
Forest plot of the secondary all-domain synthesis of VR interventions.

The single 2-month follow-up effect reported by Tabrizi et al. was g = 2.40 (95% CI 1.49 to 3.31; N = 32). This result was reported descriptively and was not combined with the immediate post-treatment effects (**Supplementary Table S4C**).

### 3.3 Exploratory technology and comparator subgroup analyses

By VR technology type, the pooled estimate was g = 0.89 (95% CI 0.33 to 1.45; k = 7) for immersive head-mounted displays, g = 0.44 (95% CI 0.16 to 0.73; k = 2) for semi-immersive VR, g = 3.27 (95% CI 2.21 to 4.33; k = 1) for non-immersive desktop VR, and g = 0.97 (95% CI 0.48 to 1.46; k = 1) for mixed reality. The conventional Wald moderator test was statistically significant (p = 0.028), whereas the Hartung–Knapp test was not (p = 0.108). Given the small and uneven numbers of studies across technology categories, these subgroup findings were considered exploratory and did not support conclusions regarding the superiority of any VR technology (**Figure 2B**; **Table 2**).

By comparator type, the pooled estimate was g = 1.25 (95% CI 0.43 to 2.06; k = 4) for active comparators, g = 0.23 (95% CI −0.08 to 0.54; k = 3) for medication/usual care, and g = 1.30 (95% CI 0.14 to 2.45; k = 4) for waitlist/no intervention. Neither the Wald nor Hartung–Knapp moderator test showed a statistically significant difference among comparator types (p = 0.257 and p = 0.344, respectively) (**Figure 2C**; **Table 2**).

### 3.4 Risk of bias and certainty of evidence

Among the 11 randomized studies assessed with RoB 2, 7 were judged at high risk of bias, 3 had some concerns, and 1 was at low risk. High-risk judgements were concentrated in bias due to deviations from intended interventions and measurement of outcomes, with 7 of 11 studies rated as high risk in each domain. All 11 studies were rated at low risk for missing outcome data and selection of the reported result. The two quasi-experimental studies were assessed separately using ROBINS-I: Tabrizi et al. was judged at moderate overall risk and Fang et al. at serious overall risk. Because RoB 2 and ROBINS-I use different judgement frameworks, their results were summarized separately rather than combined across all 13 studies (**Figure 4**; **Supplementary Table S6**, Parts A and B).

**Figure 4:**
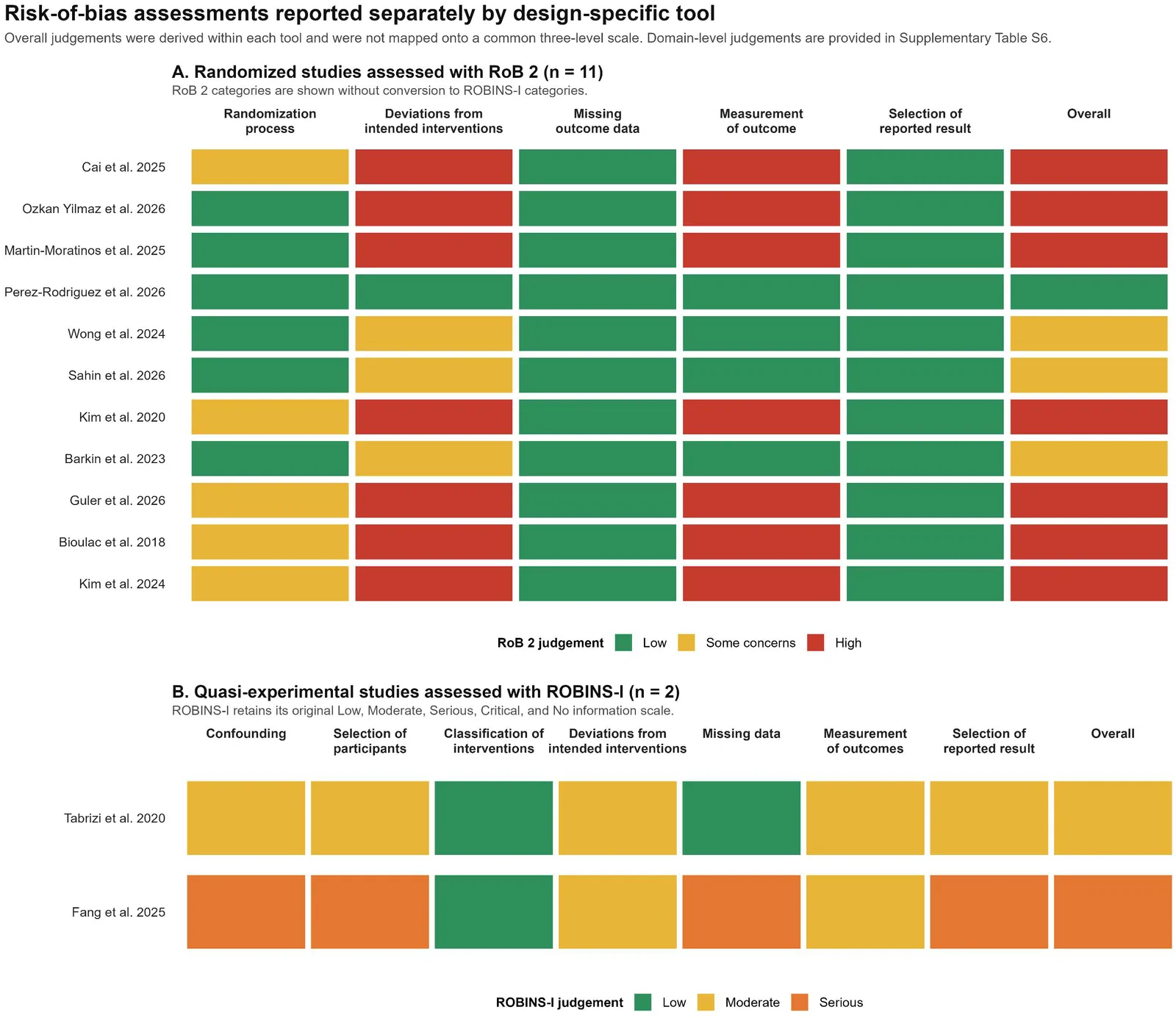
Tool-specific risk-of-bias assessments for randomized and quasi-experimental studies. **(A)** shows RoB 2 assessments for randomized studies, and **(B)** shows ROBINS-I assessments for quasi-experimental studies. Judgements were reported within each tool and were not converted to a common scale.

The certainty of evidence was rated very low for all four primary outcome domains. Downgrading reflected risk of bias, serious or very serious inconsistency, and imprecision. Risk-of-bias downgrading was based on the tool-specific RoB 2 and ROBINS-I judgements without cross-tool conversion. Publication bias was not downgraded within individual outcome domains because each included only two to seven studies; the significant Egger result from the secondary all-domain synthesis could not be attributed to any specific domain. Detailed GRADE assessments are provided in **Supplementary Table S2**, and tool-specific risk-of-bias assessments and rationales are provided in **Supplementary Table S6**, Parts A–C.

### 3.5 Sensitivity analyses, small-study effects, and meta-regression

Leave-one-study-out estimates for the secondary all-domain synthesis ranged from g = 0.79 to 1.08, with all conventional 95% confidence intervals remaining above zero. Tabrizi et al. was identified as influential by model diagnostics; omitting this study reduced the pooled estimate to g = 0.79 (95% CI 0.40 to 1.18), while substantial heterogeneity remained (I² = 85.4%) (**Figure 5**; **Table 3**).

**Figure 5:**
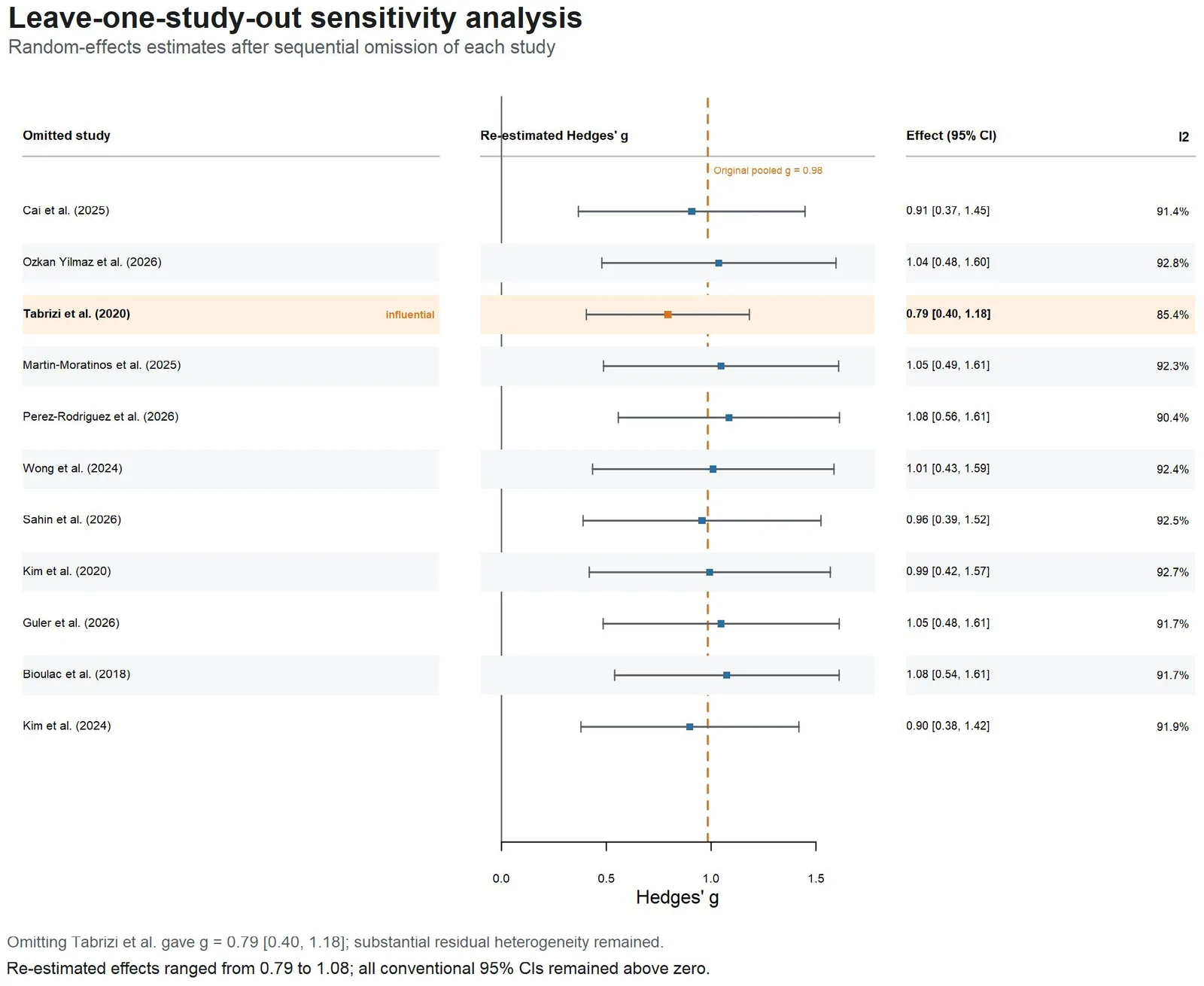
Leave-one-study-out sensitivity analysis for the secondary all-domain synthesis. Each row shows the random-effects estimate after omitting one study. The dashed vertical line indicates the original pooled estimate. Positive Hedges’ g values favor VR.

**Table 3:** Sensitivity analyses, small-study-effect diagnostics, and exploratory meta-regression.

Analysis	k	Estimate or statistic	95% CI	p value	Additional information
Leave-one-study-out range	10	g 0.79 to 1.08	All conventional CIs above 0		Sequential omission of each of 11 studies
Leave-one-out: omit Tabrizi et al.	10	g=0.79	0.40 to 1.18	<0.001	I2 = 85.4%; influential by model diagnostics
Egger regression test	11	z=2.67		0.008	Possible funnel asymmetry/small-study effects
Begg-Mazumdar rank test	11	Kendall tau=0.42		0.087	
Trim-and-fill	11	Adjusted g=0.98	0.47 to 1.50	<0.001	Imputed studies=0
Duration meta-regression: Wald z	10	beta=-0.103 g/week	-0.25 to 0.04	0.172	tau2 = 0.297
Duration meta-regression: Hartung-Knapp	10	beta=-0.103 g/week	-0.28 to 0.07	0.212	tau2 = 0.297
Duration meta-regression: Wald z; excluding midpoint-coded study	9	beta=-0.107 g/week	-0.25 to 0.03	0.139	tau2 = 0.268
Duration meta-regression: Hartung-Knapp; excluding midpoint-coded study	9	beta=-0.107 g/week	-0.28 to 0.06	0.183	tau2 = 0.268

Positive Hedges’ g values favor VR. For leave-one-study-out analyses, k = 10 denotes the number of studies retained in each refitted model; 11 omission analyses were performed. Egger and Begg–Mazumdar tests are small-study-effect diagnostics. Meta-regression coefficients (β) represent the change in Hedges’ g per additional intervention week. The primary duration analysis used the midpoint of a reported 6–8-week range, and the sensitivity analysis excluded this midpoint-coded study.

The funnel plot for the secondary all-domain synthesis was asymmetric, and Egger regression indicated possible small-study effects (z = 2.67, p = 0.008). The Begg–Mazumdar rank test was not statistically significant (Kendall’s τ = 0.42, p = 0.087), and trim-and-fill imputed no studies. Given the discordant diagnostics and the small number of studies, the observed asymmetry could not be attributed specifically to publication bias (**Figure 6**; **Table 3**).

**Figure 6:**
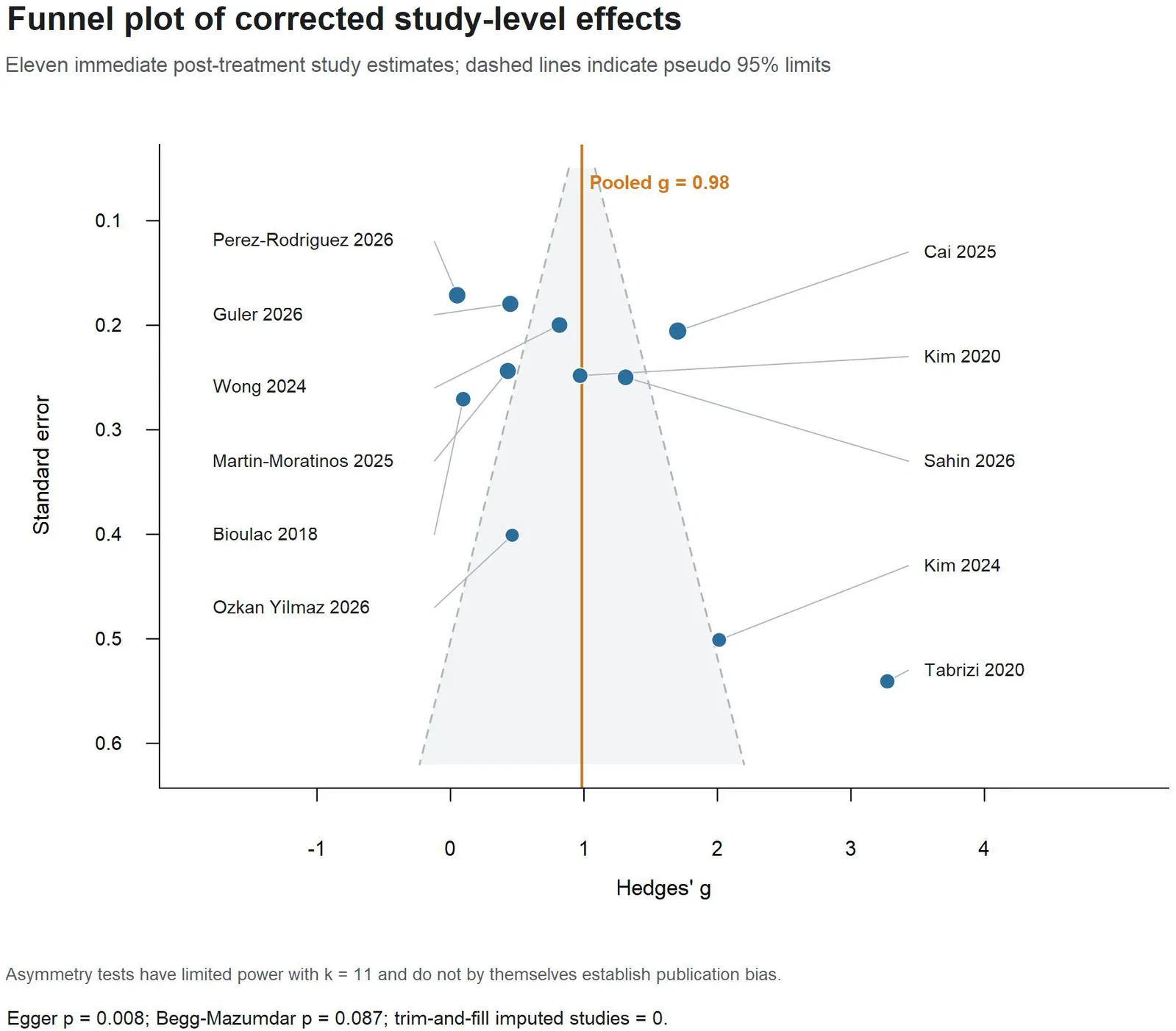
Funnel plot for the secondary all-domain synthesis. Each point represents one study-level all-domain estimate. The solid vertical line indicates the pooled random-effects estimate, and the dashed lines indicate pseudo 95% limits.

The intervention-duration meta-regression included 10 studies. The estimated slope was negative but not statistically significant (β = −0.103 Hedges’ g per week, 95% CI −0.251 to 0.045; p = 0.172), with Hartung–Knapp inference yielding p = 0.212. Excluding the study for which a reported 6–8-week duration was represented by its midpoint produced a similar estimate (β = −0.107, 95% CI −0.249 to 0.035; p = 0.139; Hartung–Knapp p = 0.183). No statistically significant association between intervention duration and effect size was observed (**Figure 7**; **Table 3**).

**Figure 7:**
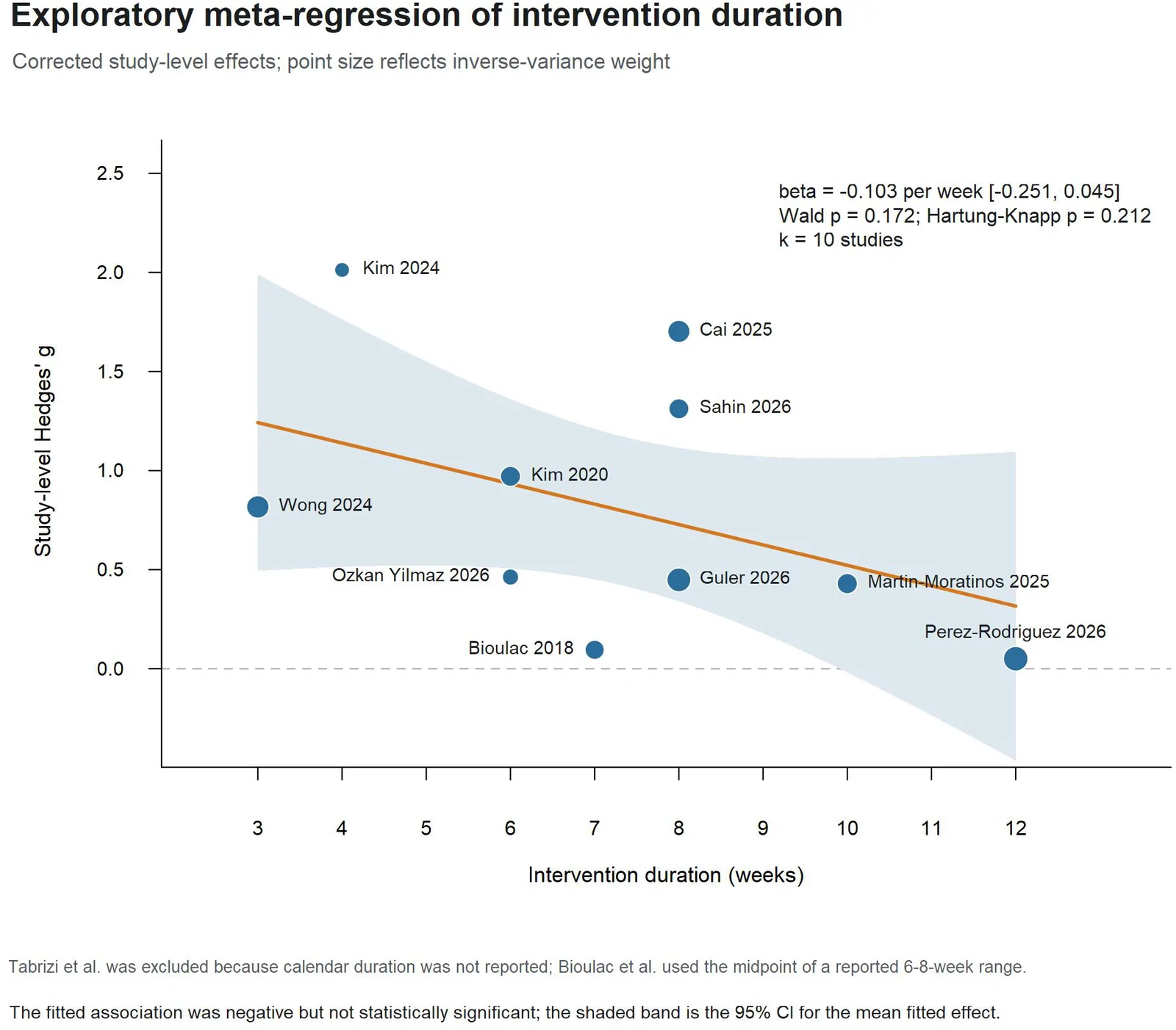
Meta-regression of intervention duration and effect size. Points represent study-level effects and are sized according to inverse-variance weights. The solid line represents the fitted random-effects meta-regression, and the shaded region represents the 95% confidence band.

### 3.6 Exploratory machine-learning analysis

The exploratory machine-learning analysis included 45 immediate post-treatment effects from 11 studies. The intercept-only benchmark yielded a study-balanced RMSE of 1.11, MAE of 0.89, and out-of-fold R² of −0.16. The sample/context ridge model yielded RMSE = 1.16, MAE = 0.96, and out-of-fold R² = −0.26, whereas the all-moderator ridge model yielded RMSE = 1.15, MAE = 0.95, and out-of-fold R² = −0.25. Both ridge models therefore showed higher prediction errors and lower out-of-fold R² than the intercept-only benchmark, providing no evidence of improved out-of-fold prediction (**Figures 8A, B**; **Supplementary Table S3A**).

**Figure 8:**
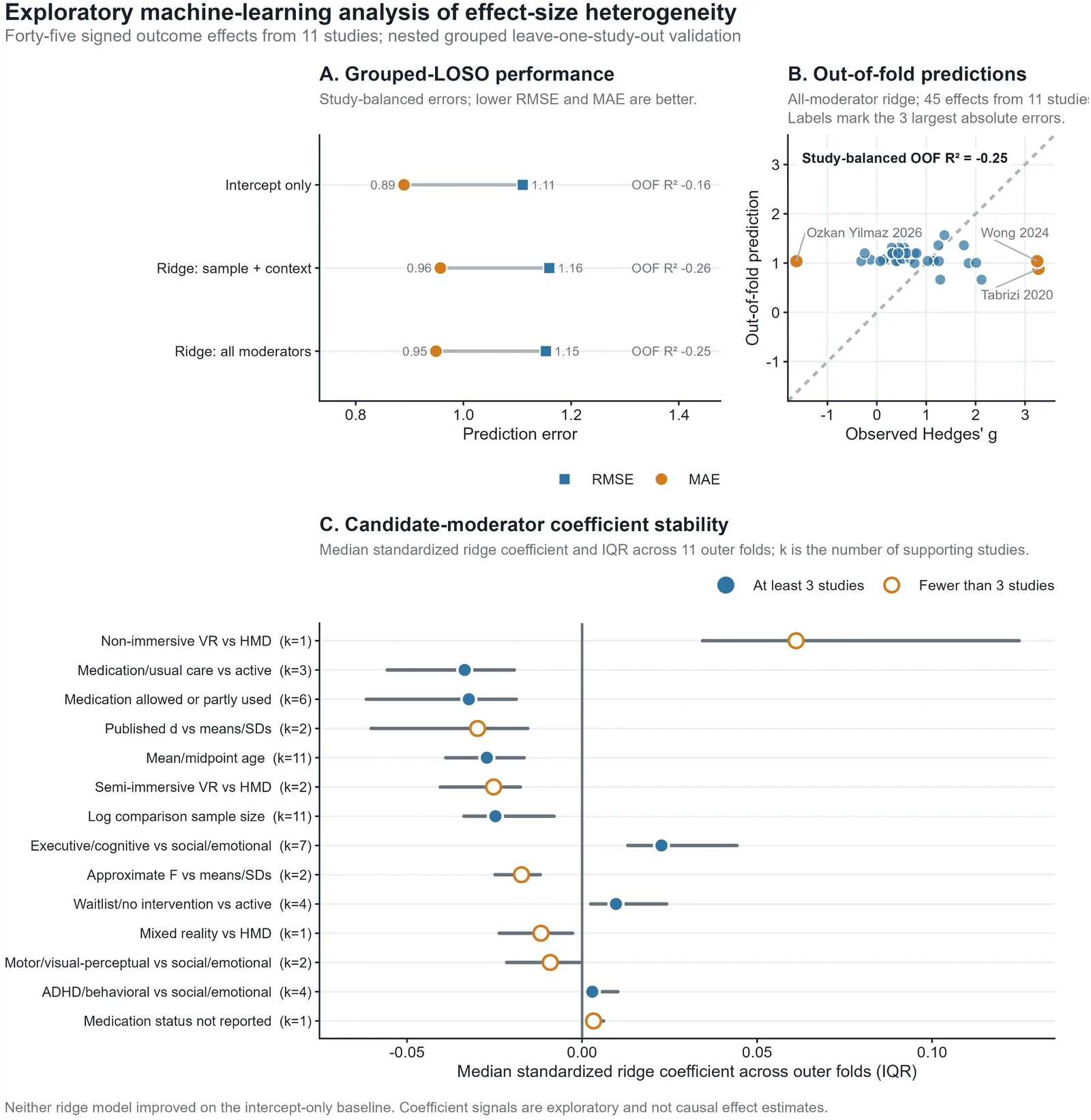
Exploratory machine-learning analysis of effect-size heterogeneity. **(A)** compares study-balanced RMSE and MAE across the intercept-only benchmark and two ridge models; lower values indicate better prediction, and out-of-fold R² is shown for each model. **(B)** shows observed versus out-of-fold predicted effects from the all-moderator ridge model; the dashed line represents perfect agreement, and labels identify the three largest absolute prediction errors. **(C)** shows median standardized ridge coefficients and interquartile ranges across the outer folds; k denotes the number of supporting studies. Filled points indicate variables supported by at least three studies and open points indicate variables supported by fewer than three studies. The intervals represent interquartile ranges rather than confidence intervals.

Median ridge coefficients were generally small and varied according to the number of supporting studies. The largest positive median coefficient was observed for non-immersive VR, although this category was represented by only one study. Other coefficients related to effect-size source, VR type, medication context, age, sample size, and outcome domain were small or supported by few studies. These coefficient patterns were therefore considered exploratory and did not identify reliable moderators of effect-size heterogeneity (**Figure 8C**; **Supplementary Table S3B**).

## 4 Discussion

Across four clinically distinct ADHD-related outcome domains, the quantitative evidence showed a positive but uneven pattern. Conventional pooled estimates favored VR for executive/cognitive, social/emotional, and motor/visual-perceptual outcomes, whereas the ADHD/behavioral estimate was imprecise and crossed the null. The executive/cognitive domain provided the clearest signal because it had the largest conventional estimate and was the only domain whose Hartung-Knapp confidence interval remained above zero. These findings suggest that VR may benefit selected functional outcomes rather than producing one uniform effect across all ADHD-related outcomes.

The robustness analyses clarified both the strength and the limits of this signal. The dependence-adjusted comparison did not identify differences among domains (p = 0.624), so the observed pattern does not establish that VR is more effective for executive/cognitive function than for other domains. The secondary all-domain estimate was positive, and its direction changed little across working correlations, multilevel modeling, and robust-variance estimation; however, substantial heterogeneity and a prediction interval spanning the null indicate that the magnitude and reproducibility of benefit may vary across outcomes and settings.

Taken together, these analyses distinguish the presence of a positive average signal from confidence in its magnitude, consistency, and generalizability. Seven of 11 randomized studies were at high risk under RoB 2, the two quasi-experimental studies had moderate and serious ROBINS-I judgements, and GRADE certainty was very low across all four domains. Subgroup, meta-regression, and machine-learning analyses did not identify a reliable explanation for heterogeneity. Thus, the evidence is most encouraging for executive/cognitive outcomes, while the overall efficacy, clinical importance, and conditions under which VR may be beneficial remain uncertain.

### 4.1 Potential mechanisms and explanatory hypotheses

The mechanisms discussed here should be regarded as explanatory hypotheses rather than pathways directly tested in the present meta-analysis. ADHD is characterized not only by core symptoms of inattention, hyperactivity, and impulsivity, but also by impairments in executive functions, including sustained attention, working memory, inhibitory control, cognitive flexibility, and goal-directed behavior (29, 30). However, executive-function deficits do not account for all manifestations of ADHD and are neither necessary nor sufficient to explain the disorder (31). Interventions that simultaneously engage multiple cognitive processes in functionally relevant contexts may therefore have the potential to influence several outcome domains, although the extent to which these effects transfer across domains remains uncertain.

One potential advantage of VR over paper-and-pencil or conventional computerized training is its ability to provide interactive and ecologically relevant environments. Evidence from cognitive training suggests that improvement on trained tasks does not necessarily generalize to core ADHD symptoms, academic performance, or everyday functioning (32). VR can simulate classroom activities, daily tasks, movement-based games, and social interactions under controlled conditions, thereby combining experimental control with greater ecological relevance (33, 34). These environments may integrate visual, auditory, cognitive, and motor demands; however, whether such features lead to reliable transfer to real-world clinical outcomes remains unclear.

VR interventions also commonly incorporate gamification, immediate feedback, task rewards, and adaptive difficulty, which may facilitate engagement in children with ADHD. Conventional cognitive training can be limited by repetitive task formats, adherence difficulties, and restricted transfer effects (35, 36). Previous meta-analytic evidence has reported positive effects of VR on attention-related outcomes in children with ADHD (37), while immersive VR and serious games have also been proposed as approaches to enhance task engagement (18). Individual studies have additionally reported potential effects on social or behavioral outcomes (38, 39). In the present review, several studies reported high session completion; however, differences in the definitions and denominators used to assess adherence prevent firm conclusions regarding the effect of VR on sustained adherence or long-term transfer.

VR may therefore be more appropriately understood as an intervention platform rather than a single treatment technique. Its effects may reflect interactions among sensory input, motor demands, cognitive processing, behavioral feedback, task characteristics, and therapist guidance, consistent with the framework of complex interventions (40). From this perspective, detailed reporting of intervention content, delivery, feedback, dose, and individualization is particularly important (41, 42). It also suggests that observed effects should not be attributed to immersion or VR hardware alone.

### 4.2 Interpretation of heterogeneity and certainty of evidence

Substantial heterogeneity limits the interpretation and generalizability of the positive domain-specific and secondary all-domain estimates. Differences in device type, training target, intensity, comparator condition, participant characteristics, and outcome measurement may all contribute. Because the prediction interval for the secondary all-domain estimate crossed the null, the positive average effect should not be assumed to apply consistently across all settings or outcomes.

First, VR technologies themselves are heterogeneous. Immersive head-mounted displays, semi-immersive VR, non-immersive desktop VR, and mixed reality differ in degree of immersion, interaction mode, feedback format, and intensity of sensory stimulation. The immersion and ecological validity of VR may influence task engagement, attentional allocation, and behavioral responses (33, 34). However, although the average estimate for immersive HMD was positive, heterogeneity within this subgroup was high (I² = 88.9%), and the technology moderator was not statistically significant under Hartung–Knapp inference. Moreover, the semi-immersive subgroup included only two studies, while the non-immersive and mixed-reality categories were each represented by a single study. These findings therefore do not establish that immersive VR is more effective than other VR technologies.

Second, variation in training content and intervention targets is another important source of heterogeneity. Some studies focused on sustained attention or response inhibition, whereas others targeted executive function, working memory, motor coordination, or social interaction. Previous reviews of serious games and VR interventions have similarly shown that ADHD-related digital interventions are highly diverse in training content and target outcomes (43, 44). Therefore, the effect sizes observed across studies may reflect differences in the training tasks themselves, rather than the influence of VR devices alone. Without clearer reporting of training modules, task progression, and feedback mechanisms, it will remain difficult to determine which components are most likely to drive treatment effects (45).

Differences in study design further increase the complexity of evidence interpretation. Included studies varied in age range, symptom severity, allowance of medication use, comparator type, training dose, and follow-up duration. For complex interventions, intervention duration, total training minutes, session intensity, and completion rate are not interchangeable (46). The duration meta-regression produced a negative coefficient but did not show a statistically significant association. Duration in weeks is also only a crude proxy for intervention dose and may be confounded with training content, comparator condition, completion rate, and study design (47). It therefore cannot be used to conclude that shorter interventions are more effective.

GRADE certainty was very low across all four primary outcome domains. This rating reflected limitations related to risk of bias, inconsistency, and imprecision and does not imply that VR is ineffective. Rather, it indicates limited confidence in the magnitude and reproducibility of the observed effects. Funnel-plot asymmetry and the significant Egger test in the secondary all-domain synthesis provide an additional contextual concern; however, the small number of studies and discordant small-study-effect diagnostics prevent this finding from being attributed specifically to publication bias or to any individual outcome domain.

### 4.3 Clinical implications of VR interventions for ADHD

From a clinical perspective, the domain-specific findings support continued evaluation of VR as an adjunctive non-pharmacological platform, with executive/cognitive outcomes representing the clearest target for confirmatory trials. Current evidence does not justify routine use of VR or its replacement of pharmacotherapy, behavioral interventions, or school-based support. Pharmacotherapy remains an important component of ADHD management, with established evidence for the control of core symptoms (48, 49). VR should therefore currently be regarded as a promising but provisional adjunct: its role in comprehensive ADHD management will depend on confirmation of domain-specific benefits in higher-quality trials.

The potential clinical value of VR lies in its capacity to provide an interactive, contextualized, and repeatable platform for non-pharmacological training. Digital interventions can deliver standardized tasks, real-time feedback, remote data recording, and dynamic difficulty adjustment. A randomized trial of a software-based digital therapeutic reported improvement in attentional performance in children with ADHD (50), although the indications, optimal dose, efficacy boundaries, and clinical role of such interventions remain to be clarified. The present review identifies functional targets for further evaluation but does not establish superiority over existing treatments or a minimum clinically important benefit.

Gamified design, immediate feedback, and potential home-based delivery may improve the engagement and accessibility of pediatric digital interventions (51). However, safety and adherence reporting in the included studies was inconsistent. Four studies used a named structured safety instrument or side-effect scale, six reported harms narratively, and three did not report study-level safety findings. Among studies reporting safety outcomes, no serious intervention-related adverse events were reported, and the reported symptoms were generally mild or transient, including dizziness, disorientation, headache or eye strain, sweating, headset discomfort, and fear of heights. Heterogeneous assessment methods and incomplete denominators prevented estimation of pooled adverse-event incidence, and lack of reporting should not be interpreted as evidence of safety. Adherence was also reported using varying definitions of attendance, completion, engagement, and attrition, while medication adherence was not systematically reported (**Supplementary Table S5**).

Finally, clinical translation of VR interventions should place greater emphasis on individualized adaptation. Children with ADHD differ substantially in age, symptom profile, cognitive deficits, comorbidities, and family support; therefore, a single VR training program is unlikely to be suitable for all children. In future clinical applications, different training modules should be selected according to each child’s primary functional impairment, such as sustained attention training, inhibitory control training, classroom simulation, social interaction training, or motor coordination training. At the same time, VR systems should retain professional supervision and treatment-effect monitoring to avoid being reduced to home entertainment tools without clinical oversight. Only after indications, training dose, safety, and real-world effectiveness are further clarified can VR become a scalable adjunctive intervention within comprehensive ADHD management.

### 4.4 Methodological insights into understanding treatment heterogeneity

An important methodological feature of this review was the explicit separation of clinically distinct outcome domains before synthesis. This approach preserves the clinical meaning of ADHD/behavioral, executive/cognitive, social/emotional, and motor/visual-perceptual outcomes, while the secondary all-domain composite provides only broad descriptive context. Such separation is particularly relevant for complex digital interventions, for which effects may differ across intervention targets and implementation settings. The machine-learning analysis complemented this framework by exploring heterogeneity after effect-size estimation; it neither replaced the domain-specific meta-analyses nor contributed to their pooled estimates. Accordingly, explanations involving immersion, engagement, or training content were treated as hypotheses regarding heterogeneity rather than substitutes for the observed domain-specific estimates.

Meta-regression and exploratory machine learning extended the domain-specific syntheses by examining whether the observed heterogeneity could be explained or predicted using available study-level characteristics. Neither approach identified reliable moderator evidence, which may partly reflect the limited number of studies and the lack of consistently reported study-level characteristics. Future trials should therefore prospectively and consistently report candidate sources of heterogeneity, including intervention content, technology type, dose, medication context, participant characteristics, and outcome selection.

This finding reflects an important problem in the current literature on VR interventions for ADHD: intervention descriptions and data reporting remain insufficient. Existing studies usually report VR device type and intervention duration, but provide limited information on key factors such as specific training modules, task progression strategies, feedback mechanisms, reward methods, completion rates, dropout rates, family involvement, and therapist guidance. Both TIDieR and its rehabilitation extension emphasize that complex intervention studies should fully report intervention content, delivery mode, dose, individualization, and adherence to support interpretation and replication (41, 42). Therefore, the main limitation in explaining treatment effects may lie not in the statistical methods themselves, but in the quality of study design and reporting. Future randomized trials should also strengthen preregistration, blinded outcome assessment, intention-to-treat analysis, and standardized outcome reporting in accordance with current trial-reporting guidance (52).

Exploratory machine learning provided an additional assessment of whether the available study-level characteristics could predict effect-size variation across studies. Neither ridge model improved out-of-study prediction relative to the intercept-only benchmark, and the estimated coefficients were based on few study clusters and sparsely represented categories. Thus, the available aggregate study-level characteristics were insufficient to identify reproducible predictors of effect-size heterogeneity. Larger evidence bases with prospectively standardized variables, and potentially individual participant data, will be needed to determine whether reproducible effect modifiers can be identified (53–55).

### 4.5 Limitations

This study has several limitations. First, each primary outcome domain included only two to seven studies, and some technology subgroups were represented by only one study, limiting the precision of domain-specific and subgroup estimates. Second, risk of bias remained important: 7 of 11 randomized studies were judged at high risk under RoB 2, while the two quasi-experimental studies were judged at moderate and serious risk under ROBINS-I. Third, substantial clinical and statistical heterogeneity remained within several outcome domains, and the secondary all-domain synthesis combined clinically distinct outcome constructs and should therefore be interpreted descriptively. Fourth, incomplete reporting of study-level characteristics limited robust moderator analyses, while multiple outcomes within individual studies introduced statistical dependence that could only be addressed through assumed covariance structures and sensitivity analyses. Finally, long-term follow-up data were sparse, and safety and adherence reporting remained heterogeneous, with inconsistent assessment methods and no common definition of adherence.

## 5 Conclusion

Current evidence suggests preliminary positive signals for VR interventions across several ADHD-related outcome domains, with the clearest evidence observed for executive/cognitive outcomes. Positive conventional estimates were also observed for social/emotional and motor/visual-perceptual outcomes, whereas the effect on ADHD/behavioral symptoms remained uncertain. However, substantial heterogeneity, a prediction interval spanning the null, important risk of bias, and very low GRADE certainty limit confidence in the magnitude, consistency, and generalizability of these findings. VR should therefore be regarded as a promising adjunctive approach for further investigation rather than an intervention with established routine clinical efficacy. Current evidence does not demonstrate superiority among outcome domains or identify reliable moderators of treatment-effect heterogeneity.

Future confirmatory trials should prioritize domain-specific and clinically meaningful outcomes, adequate sample sizes, rigorous risk-of-bias control, standardized reporting of intervention content and safety, and longer-term follow-up. More consistent prospective reporting of intervention components, participant characteristics, treatment dose, and implementation conditions will also be needed to clarify when, for whom, and under what circumstances VR interventions may provide clinically meaningful benefit.

## Data Availability

The original contributions presented in the study are included in the article/**Supplementary Material**. Further inquiries can be directed to the corresponding authors.
