# Supplementary material for: Effects of VR interventions on pediatric ADHD: a systematic review, meta-analysis and exploratory machine-learning analysis
Source: Front Psychiatry. 2026 Sep 30;17:1936884. doi: 10.3389/fpsyt.2026.1936884 (PMC13664293; doi:10.3389/fpsyt.2026.1936884)
Supplement: Supplementary file 1 [file Table1.docx]

**Supplementary Table S1. Keyword-based search strategy and database-specific adaptations**

| **Database** | **Language** | **Core keywords used** | **Database-specific adaptation / search note** |
| --- | --- | --- | --- |
| PubMed | English | virtual reality; VR; attention-deficit/hyperactivity disorder; ADHD; child; children; adolescent; adolescents; pediatric | Keyword-based searches were conducted using combinations of VR-related terms, ADHD-related terms, and child/adolescent-related terms. Searches were adapted to PubMed keyword/title/abstract retrieval functions. |
| Embase | English | virtual reality; VR; attention-deficit/hyperactivity disorder; ADHD; child; children; adolescent; adolescents; pediatric | Keyword-based searches were conducted using combinations of the three core concepts: virtual reality, ADHD, and children/adolescents. Keyword-based searches were adapted to the Embase retrieval interface. No additional Emtree expansion was reported. |
| Web of Science Core Collection | English | virtual reality; VR; ADHD; attention-deficit/hyperactivity disorder; child; children; adolescent; adolescents | Searches were performed using topic/keyword fields, combining VR-related, ADHD-related, and pediatric population-related keywords. |
| IEEE Xplore | English | virtual reality; VR; ADHD; attention-deficit/hyperactivity disorder; children; adolescents | Because IEEE Xplore is a technology-oriented database, searches focused on VR-related and ADHD-related terms combined with pediatric population keywords. |
| Cochrane Central Register of Controlled Trials CENTRAL | English | virtual reality; VR; ADHD; attention-deficit/hyperactivity disorder; child; adolescent; pediatric | Keyword-based searches were used to identify controlled trials or intervention studies involving VR and ADHD in children or adolescents. |
| ScienceDirect | English | virtual reality; VR; ADHD; attention-deficit/hyperactivity disorder; children; adolescents; pediatric | Searches were conducted using keyword combinations related to VR intervention, ADHD, and pediatric populations. |
| CNKI | Chinese and English | 虚拟现实；VR；注意缺陷多动障碍；多动症；ADHD；儿童；青少年 | Chinese keyword-based searches were conducted using VR-related terms, ADHD-related terms, and child/adolescent-related terms. English abbreviations such as VR and ADHD were also used when applicable. |

Note: The search was conducted using keyword-based queries rather than a fully prespecified Boolean search string. Keywords were organized around three core concepts: virtual reality, ADHD, and children/adolescents. Database-specific adaptations were made according to the retrieval functions of each platform. Searches were limited to English and Chinese publications.

注：本研究采用关键词式检索，而非预先设定的完整布尔检索式。检索词围绕三个核心概念展开：虚拟现实、ADHD以及儿童/青少年。各数据库根据平台检索功能进行适当调整，检索语言限定为英文和中文。
