# Supplementary material for: Effects of VR interventions on pediatric ADHD: a systematic review, meta-analysis and exploratory machine-learning analysis
Source: Front Psychiatry. 2026 Sep 30;17:1936884. doi: 10.3389/fpsyt.2026.1936884 (PMC13664293; doi:10.3389/fpsyt.2026.1936884)
Supplement: Supplementary file 2 [file Table2.docx]

**Supplementary Table S2. GRADE certainty of evidence for immediate post-treatment effects of virtual reality interventions across the four primary outcome domains**

Positive Hedges' g values favour virtual reality. Certainty was assessed separately for each primary outcome domain using the five GRADE domains.

| **Outcome domain** | **Evidence body** | **Hedges' g [95% CI]** | **Risk of bias** | **Inconsistency** | **Indirectness** | **Imprecision** | **Publication bias** | **Certainty** |
| --- | --- | --- | --- | --- | --- | --- | --- | --- |
| ADHD/behavioral symptoms | k=4; N=229 4 randomized trials | 0.49 [-0.59, 1.57] | Serious (-1)^a^ | Very serious (-2)^b^ | Not serious (0)^c^ | Very serious (-2)^d^ | Not separately downgraded (0)^e^ | Very low (⊕◯◯◯) |
| Executive/cognitive function | k=7; N=341 6 randomized trials; 1 quasi-experimental study | 1.15 [0.38, 1.92] | Serious (-1)^a^ | Serious (-1)^b^ | Not serious (0)^c^ | Serious (-1)^d^ | Not separately downgraded (0)^e^ | Very low (⊕◯◯◯) |
| Social/emotional function | k=4; N=225 4 randomized trials | 0.51 [0.08, 0.93] | Serious (-1)^a^ | Serious (-1)^b^ | Not serious (0)^c^ | Serious (-1)^d^ | Not separately downgraded (0)^e^ | Very low (⊕◯◯◯) |
| Motor/visual-perceptual function | k=2; N=100 2 randomized trials | 0.88 [0.02, 1.74] | Serious (-1)^a^ | Serious (-1)^b^ | Not serious (0)^c^ | Very serious (-2)^d^ | Not separately downgraded (0)^e^ | Very low (⊕◯◯◯) |

**^a^ Risk of bias.** Tool-specific judgements were retained without cross-tool mapping. ADHD/behavioural symptoms: among four RoB 2 studies, 3 were high risk and 1 was low risk; high-risk studies contributed 74.0% of random-effects weight. Executive/cognitive function: among six RoB 2 studies, 4 were high risk, 1 had some concerns, and 1 was low risk; RoB 2 high-risk studies contributed 57.3% of weight. The additional quasi-experimental study was moderate risk under ROBINS-I. Social/emotional function: among four RoB 2 studies, 2 were high risk, 1 had some concerns, and 1 was low risk; high-risk studies contributed 41.6% of weight. Motor/visual-perceptual function: both studies used RoB 2; 1 was high risk and 1 had some concerns.

**^b^ Inconsistency.** Heterogeneity and 95% prediction intervals were: ADHD/behavioural symptoms, I2=93.5%, -1.85 to 2.83; executive/cognitive function, I2=93.5%, -0.92 to 3.22; social/emotional function, I2=60.0%, -0.26 to 1.28; and motor/visual-perceptual function, I2=82.9%, -0.52 to 2.28. ADHD/behavioural estimates also differed in direction, supporting a two-level downgrade.

**^c^ Indirectness.** Not downgraded because the pediatric ADHD populations, VR interventions, comparators, and domain-specific immediate post-treatment outcomes addressed the review question.

**^d^ Imprecision.** No validated common minimally important difference was available across scales. Judgements considered confidence-interval width, Hartung-Knapp sensitivity intervals, and an approximate optimal information size of 800 participants for an SMD of 0.20 (two-sided alpha=0.05; 80% power). Hartung-Knapp intervals were -1.25 to 2.23, 0.16 to 2.14, -0.21 to 1.22, and -4.68 to 6.44, respectively.

**^e^ Publication bias.** Outcome-specific tests were not considered reliable because each domain contained only 2-7 studies. The shared 11-study all-domain evidence base showed funnel asymmetry and an Egger small-study-effect signal (p=0.008), but this signal could not be attributed to any individual outcome domain and was not used for a separate downgrade. It remains a contextual concern rather than proof of publication bias.

**Scope note.** No formal certainty rating was assigned to the secondary all-domain composite, which combines clinically distinct constructs, or to exploratory technology and comparator subgroups. Analysis-set N denotes participants in selected pairwise comparisons or reported analysis sets and is not additive across rows. Very low certainty means that the evidence is very uncertain about the true effect; it does not mean that the intervention has no effect.

Abbreviations: ADHD, attention-deficit/hyperactivity disorder; CI, confidence interval; GRADE, Grading of Recommendations Assessment, Development and Evaluation; I2, proportion of heterogeneity; RoB 2, Cochrane Risk of Bias 2; ROBINS-I, Risk Of Bias In Non-randomized Studies of Interventions; SMD, standardized mean difference; VR, virtual reality.
