# Supplementary material for: Effects of VR interventions on pediatric ADHD: a systematic review, meta-analysis and exploratory machine-learning analysis
Source: Front Psychiatry. 2026 Sep 30;17:1936884. doi: 10.3389/fpsyt.2026.1936884 (PMC13664293; doi:10.3389/fpsyt.2026.1936884)
Supplement: Supplementary file 3 [file Table3.docx]

Supplementary Table S3a. Grouped leave-one-study-out machine-learning performance

| **Model** | **Outcomes** | **Studies** | **Study balanced RMSE** | **Study balanced MAE** | **Study balanced OOF R2** | **Unweighted RMSE** | **Unweighted MAE** | **Unweighted OOF R2** |
| --- | --- | --- | --- | --- | --- | --- | --- | --- |
| Intercept only | 45 | 11 | 1.11 | 0.89 | -0.16 | 0.98 | 0.79 | -0.29 |
| Ridge: sample + context | 45 | 11 | 1.16 | 0.96 | -0.26 | 1.03 | 0.86 | -0.44 |
| Ridge: all candidate moderators | 45 | 11 | 1.15 | 0.95 | -0.25 | 1.04 | 0.86 | -0.45 |

Note. Performance was evaluated using nested grouped leave-one-study-out validation with study-balanced inverse-variance weights. RMSE and MAE are lower-is-better error metrics. Out-of-fold R2 compares prediction error with the held-out-data mean benchmark. Neither ridge model outperformed the intercept-only model; the candidate variables therefore did not improve grouped out-of-fold prediction.

Supplementary Table S3b. Candidate moderator coefficient stability

| **Candidate moderator** | **Median coefficient** | **IQR** | **Sign consistency** | **Supporting outcomes** | **Supporting studies** | **Low support** |
| --- | --- | --- | --- | --- | --- | --- |
| Non-immersive VR (vs immersive HMD) | 0.061 | 0.03 to 0.12 | 0.91 | 1 | 1 | Yes |
| Medication/usual care (vs active) | -0.034 | -0.06 to -0.02 | 1.00 | 12 | 3 | No |
| Medication allowed or partly used | -0.032 | -0.06 to -0.02 | 1.00 | 31 | 6 | No |
| Published d (vs means/SDs) | -0.030 | -0.06 to -0.02 | 1.00 | 12 | 2 | Yes |
| Mean/midpoint age | -0.027 | -0.04 to -0.02 | 1.00 | 45 | 11 | No |
| Semi-immersive VR (vs immersive HMD) | -0.025 | -0.04 to -0.02 | 1.00 | 12 | 2 | Yes |
| Log comparison sample size | -0.025 | -0.03 to -0.01 | 1.00 | 45 | 11 | No |
| Executive/cognitive (vs social/emotional) | 0.023 | 0.01 to 0.04 | 1.00 | 25 | 7 | No |
| Approximate F conversion (vs means/SDs) | -0.017 | -0.02 to -0.01 | 1.00 | 6 | 2 | Yes |
| Waitlist/no intervention (vs active) | 0.010 | 0.00 to 0.02 | 0.82 | 23 | 4 | No |
| Mixed reality (vs immersive HMD) | -0.012 | -0.02 to -0.00 | 0.91 | 5 | 1 | Yes |
| Motor/visual-perceptual (vs social/emotional) | -0.009 | -0.02 to -0.00 | 0.82 | 5 | 2 | Yes |
| ADHD/behavioral (vs social/emotional) | 0.003 | 0.00 to 0.01 | 0.82 | 7 | 4 | No |
| Medication status not reported | 0.003 | 0.00 to 0.01 | 0.91 | 2 | 1 | Yes |

Note. Coefficients are fold-specific standardized ridge estimates summarized by the median and interquartile range across outer study folds. Low support denotes fewer than three contributing studies. Coefficients are shrinkage-dependent exploratory descriptions and should not be interpreted as reliable moderators, causal effects, validated predictive factors, or a basis for treatment selection.
