# Supplementary material for: Effects of VR interventions on pediatric ADHD: a systematic review, meta-analysis and exploratory machine-learning analysis
Source: Front Psychiatry. 2026 Sep 30;17:1936884. doi: 10.3389/fpsyt.2026.1936884 (PMC13664293; doi:10.3389/fpsyt.2026.1936884)
Supplement: Supplementary file 4 [file Table4.docx]

**Supplementary Table S4. Dependency, effect-source, and time-point sensitivity analyses**

The primary working within-study correlation was rho=0.50. All analyses use signed immediate post-treatment effects unless explicitly identified as a later follow-up.

**Supplementary Table S4a. Sensitivity to the assumed within-study correlation**

| **Analysis** | **rho** | **k** | **Pairwise N** | **Hedges g** | **95% CI** | **p value** | **I2 percent** | **tau2** |
| --- | --- | --- | --- | --- | --- | --- | --- | --- |
| ADHD/behavioral symptoms | 0.00 | 4 | 229 | 0.51 | -0.57 to 1.58 | 0.355 | 94.9 | 1.125 |
| ADHD/behavioral symptoms | 0.25 | 4 | 229 | 0.50 | -0.58 to 1.58 | 0.365 | 94.1 | 1.124 |
| ADHD/behavioral symptoms | 0.50 | 4 | 229 | 0.49 | -0.59 to 1.57 | 0.374 | 93.5 | 1.122 |
| ADHD/behavioral symptoms | 0.75 | 4 | 229 | 0.48 | -0.60 to 1.57 | 0.383 | 93.0 | 1.120 |
| ADHD/behavioral symptoms | 1.00 | 4 | 229 | 0.48 | -0.61 to 1.57 | 0.391 | 92.6 | 1.118 |
| Executive/cognitive function | 0.00 | 7 | 341 | 1.14 | 0.38 to 1.90 | 0.003 | 97.3 | 0.957 |
| Executive/cognitive function | 0.25 | 7 | 341 | 1.15 | 0.38 to 1.91 | 0.003 | 95.1 | 0.959 |
| Executive/cognitive function | 0.50 | 7 | 341 | 1.15 | 0.38 to 1.92 | 0.003 | 93.5 | 0.961 |
| Executive/cognitive function | 0.75 | 7 | 341 | 1.16 | 0.38 to 1.93 | 0.003 | 92.0 | 0.963 |
| Executive/cognitive function | 1.00 | 7 | 341 | 1.16 | 0.38 to 1.94 | 0.003 | 90.7 | 0.965 |
| Motor/visual-perceptual function | 0.00 | 2 | 100 | 0.87 | 0.01 to 1.73 | 0.047 | 90.1 | 0.345 |
| Motor/visual-perceptual function | 0.25 | 2 | 100 | 0.87 | 0.02 to 1.73 | 0.046 | 86.5 | 0.331 |
| Motor/visual-perceptual function | 0.50 | 2 | 100 | 0.88 | 0.02 to 1.74 | 0.045 | 82.9 | 0.317 |
| Motor/visual-perceptual function | 0.75 | 2 | 100 | 0.88 | 0.02 to 1.74 | 0.044 | 79.3 | 0.304 |
| Motor/visual-perceptual function | 1.00 | 2 | 100 | 0.89 | 0.03 to 1.74 | 0.043 | 75.8 | 0.290 |
| Secondary all-domain descriptive summary | 0.00 | 11 | 584 | 0.97 | 0.47 to 1.47 | <0.001 | 96.3 | 0.655 |
| Secondary all-domain descriptive summary | 0.25 | 11 | 584 | 0.98 | 0.47 to 1.48 | <0.001 | 93.8 | 0.656 |
| Secondary all-domain descriptive summary | 0.50 | 11 | 584 | 0.98 | 0.47 to 1.50 | <0.001 | 91.7 | 0.658 |
| Secondary all-domain descriptive summary | 0.75 | 11 | 584 | 0.99 | 0.47 to 1.51 | <0.001 | 89.7 | 0.660 |
| Secondary all-domain descriptive summary | 1.00 | 11 | 584 | 1.00 | 0.48 to 1.52 | <0.001 | 87.9 | 0.663 |
| Social/emotional function | 0.00 | 4 | 225 | 0.49 | 0.02 to 0.96 | 0.042 | 76.1 | 0.169 |
| Social/emotional function | 0.25 | 4 | 225 | 0.50 | 0.05 to 0.95 | 0.029 | 68.4 | 0.138 |
| Social/emotional function | 0.50 | 4 | 225 | 0.51 | 0.08 to 0.93 | 0.019 | 60.0 | 0.108 |
| Social/emotional function | 0.75 | 4 | 225 | 0.51 | 0.11 to 0.90 | 0.012 | 50.0 | 0.079 |
| Social/emotional function | 1.00 | 4 | 225 | 0.51 | 0.14 to 0.87 | 0.006 | 37.6 | 0.051 |

Note. The primary working correlation was rho=0.50. The correlation was assumed rather than estimated and was varied from 0 to 1. N denotes participants in selected pairwise comparisons and is not additive across outcome-domain rows.

**Supplementary Table S4b. Dependence-aware and small-sample sensitivity models**

| **Analysis** | **Method** | **Studies** | **Effects** | **Hedges g** | **95% CI** | **p value** | **df** |
| --- | --- | --- | --- | --- | --- | --- | --- |
| ADHD/behavioral symptoms | Hartung-Knapp | 4 |  | 0.49 | -1.25 to 2.23 | 0.436 |  |
| Executive/cognitive function | Hartung-Knapp | 7 |  | 1.15 | 0.16 to 2.14 | 0.029 |  |
| Motor/visual-perceptual function | Hartung-Knapp | 2 |  | 0.88 | -4.68 to 6.44 | 0.294 |  |
| Social/emotional function | Hartung-Knapp | 4 |  | 0.51 | -0.21 to 1.22 | 0.111 |  |
| Secondary all-domain descriptive summary | Hartung-Knapp | 11 |  | 0.98 | 0.37 to 1.60 | 0.005 |  |
| ADHD/behavioral symptoms | Multilevel random-effects model | 4 | 7 | 0.51 | -0.56 to 1.59 | 0.348 |  |
| Social/emotional function | Multilevel random-effects model | 4 | 8 | 0.57 | -0.37 to 1.52 | 0.233 |  |
| Executive/cognitive function | Multilevel random-effects model | 7 | 25 | 1.13 | 0.36 to 1.89 | 0.004 |  |
| Motor/visual-perceptual function | Multilevel random-effects model | 2 | 5 | 0.85 | 0.02 to 1.67 | 0.045 |  |
| Secondary all-domain descriptive summary | Multilevel random-effects model | 11 | 45 | 0.86 | 0.47 to 1.25 | <0.001 |  |
| ADHD/behavioral symptoms | Correlated-effects RVE | 4 | 7 | 0.48 | -1.29 to 2.25 | 0.451 | 2.99 |
| Social/emotional function | Correlated-effects RVE | 4 | 8 | 0.45 | -0.29 to 1.18 | 0.149 | 2.96 |
| Executive/cognitive function | Correlated-effects RVE | 7 | 25 | 1.13 | 0.19 to 2.08 | 0.026 | 5.93 |
| Motor/visual-perceptual function | Correlated-effects RVE | 2 | 5 | 0.88 | -4.68 to 6.44 | 0.294 | 1.00 |
| Secondary all-domain descriptive summary | Correlated-effects RVE | 11 | 45 | 1.05 | 0.47 to 1.62 | 0.002 | 9.90 |

Note. Hartung-Knapp models used study-domain or study-level composites. Multilevel and correlated-effects RVE models retained outcome-level effects and clustered them by study. RVE used a small-sample correction.

**Supplementary Table S4c. Effect-source and time-point sensitivity analyses**

| **Analysis** | **k** | **Pairwise N** | **Hedges g** | **95% CI** | **p value** | **Interpretation** |
| --- | --- | --- | --- | --- | --- | --- |
| ADHD/behavioral symptoms: source-restriction sensitivity | 3 | 213 | 0.30 | -1.10 to 1.70 | 0.676 | Excluded study-domain composites containing approximate F conversions |
| Executive/cognitive function: source-restriction sensitivity | 7 | 341 | 1.15 | 0.38 to 1.92 | 0.003 | Excluded study-domain composites containing approximate F conversions |
| Motor/visual-perceptual function: source-restriction sensitivity | 2 | 100 | 0.88 | 0.02 to 1.74 | 0.045 | Excluded study-domain composites containing approximate F conversions |
| Social/emotional function: source-restriction sensitivity | 2 | 140 | 0.70 | 0.06 to 1.35 | 0.033 | Excluded study-domain composites containing approximate F conversions |
| Secondary all-domain descriptive summary: source-restriction sensitivity | 9 | 499 | 1.11 | 0.50 to 1.73 | <0.001 | Excluded all studies containing approximate F conversions |
| Tabrizi et al. 2-month follow-up (descriptive only) | 1 | 32 | 2.40 | 1.49 to 3.31 |  | Excluded from all immediate post-treatment pools |

Note. Source-restriction analyses excluded composites containing approximate conversions from F statistics. The Tabrizi et al. 2-month effect was summarized descriptively and was excluded from all immediate post-treatment pools.
