# Supplementary material for: Effects of VR interventions on pediatric ADHD: a systematic review, meta-analysis and exploratory machine-learning analysis
Source: Front Psychiatry. 2026 Sep 30;17:1936884. doi: 10.3389/fpsyt.2026.1936884 (PMC13664293; doi:10.3389/fpsyt.2026.1936884)
Supplement: Supplementary file 5 [file Table5.docx]

**Supplementary Table S5. Study-level reporting of quantitative-synthesis eligibility, adherence, adverse events, and cybersickness**

| **Study** | **Quantitative synthesis** | **Adherence/retention** | **Safety/adverse events** | **Cybersickness assessment/reporting** |
| --- | --- | --- | --- | --- |
| Cai et al. 2025 | Yes; 2 retained effects | NR | NR | No named cybersickness instrument or study-level finding identified |
| Ozkan Yilmaz et al. 2026 | Yes; 5 retained effects | No numerical attendance or completion rate reported | Authors reported no adverse events | Simulator Sickness Questionnaire (SSQ), VR group n=8: discomfort was generally low; disorientation was higher and nausea lowest; all but two participants had low overall discomfort |
| Tabrizi et al. 2020 | Yes; 1 retained effect | NR | NR | No named cybersickness instrument or study-level finding identified |
| Martin-Moratinos et al. 2025 | Yes; 1 retained effect | 76 randomized; 7 dropouts (5 VR, 2 control); all participants completed 10 face-to-face sessions; 57/76 completed at least 16 sessions (80%) | No session interruption or event lasting more than 1 h; headset discomfort 2/33, fatigue 8/33, boredom 4/33, and dizziness 2/33; no clinically significant adverse event | Structured UKU side-effect assessment; symptoms and headset discomfort reported |
| Perez-Rodriguez et al. 2026 | Yes; 6 retained effects | Adherence defined as at least 80%; 35/38 intervention participants (92.1%) met the threshold; 1/38 withdrew (2.6%) | No intervention-related adverse events reported | No named cybersickness instrument identified |
| Wong et al. 2024 | Yes; 6 retained effects | All 30 VR participants attended all 12 sessions; 1 traditional-SST participant and 11 waitlist participants withdrew | No discomfort, fatigue, headache, eyestrain, nausea, concentration difficulty, blurred vision, or dizziness; sweating reported by 16/30 VR participants | SSQ administered to the VR group |
| Sahin et al. 2026 | Yes; 2 retained effects | All 60 randomized participants completed the study | No serious adverse event; 2/30 VR participants reported mild transient dizziness in the first 1-2 sessions, without withdrawal | No named cybersickness instrument identified |
| Kim et al. 2020 | Yes; 5 retained effects | All 20 intervention participants completed the post-session survey | All SSQ symptoms were absent except fullness/heaviness of the head in one participant, attributed to HoloLens weight | SSQ administered after the intervention |
| Barkin et al. 2023 | No (a): both arms used the same tablet game; contrast isolated therapist guidance | 176 randomized; no missing data reported | No adverse events before/during data collection or intervention | No named cybersickness instrument reported |
| Guler et al. 2026 | Yes; 11 retained effects | 70 participants were included and pre/post assessments were reported; no common adherence threshold | Mild headache or eye strain requiring short breaks; number affected not reported | No named cybersickness instrument identified |
| Fang et al. 2025 | No (b): reward-enhanced VR was compared with VR without feedback; contrast isolated reward feedback | NR | NR | No named cybersickness instrument or study-level finding identified |
| Bioulac et al. 2018 | Yes; 5 retained effects | All participants in the virtual-classroom group completed training | No cybersickness-related side effects reported in the virtual-classroom group | Narrative cybersickness reporting; no named instrument identified |
| Kim et al. 2024 | Yes; 1 retained effect | All 12 intervention participants completed eight sessions | One participant attempted to remove the HMD because of fear of heights but completed the exercise with reassurance | No formal adverse-event or cybersickness instrument identified |

Note. NR, not reported at study level; SSQ, Simulator Sickness Questionnaire; SST, social-skills training; UKU, UKU Side Effect Rating Scale. NR was not interpreted as absence of an event. (a) Barkin et al.: both groups used the same Brawl Stars game on a 7.9-inch iPad; the contrast isolated therapist guidance rather than VR exposure. (b) Fang et al.: reward-enhanced VR was compared with conventional VR without feedback; the contrast isolated reward feedback rather than VR exposure. Safety findings were not pooled because ascertainment methods, denominators, and definitions differed across studies.
