# Supplementary material for: Effects of VR interventions on pediatric ADHD: a systematic review, meta-analysis and exploratory machine-learning analysis
Source: Front Psychiatry. 2026 Sep 30;17:1936884. doi: 10.3389/fpsyt.2026.1936884 (PMC13664293; doi:10.3389/fpsyt.2026.1936884)
Supplement: Supplementary file 6 [file Table6.docx]

**Supplementary Table S6. Tool-specific risk-of-bias assessments**

RoB 2 and ROBINS-I judgements are reported on their original scales. No cross-tool relabelling or pooled percentage is used.

**Part A. Randomized studies assessed with RoB 2**

| **Study** | **Randomization process** | **Deviations from intended interventions** | **Missing outcome data** | **Measurement of outcome** | **Selection of reported result** | **Overall** |
| --- | --- | --- | --- | --- | --- | --- |
| Cai et al. 2025 | Some concerns | High | Low | High | Low | High |
| Ozkan Yilmaz et al. 2026 | Low | High | Low | High | Low | High |
| Martin-Moratinos et al. 2025 | Low | High | Low | High | Low | High |
| Perez-Rodriguez et al. 2026 | Low | Low | Low | Low | Low | Low |
| Wong et al. 2024 | Low | Some concerns | Low | Low | Low | Some concerns |
| Sahin et al. 2026 | Low | Some concerns | Low | Low | Low | Some concerns |
| Kim et al. 2020 | Some concerns | High | Low | High | Low | High |
| Barkin et al. 2023 | Low | Some concerns | Low | Low | Low | Some concerns |
| Guler et al. 2026 | Some concerns | High | Low | High | Low | High |
| Bioulac et al. 2018 | Some concerns | High | Low | High | Low | High |
| Kim et al. 2024 | Some concerns | High | Low | High | Low | High |

Note. RoB 2 uses Low risk, Some concerns, and High risk. Overall judgements were derived within RoB 2 and were not translated into ROBINS-I categories.

**Part B. Quasi-experimental studies assessed with ROBINS-I**

| **Study** | **Confounding** | **Selection of participants** | **Classification of interventions** | **Deviations from intended interventions** | **Missing data** | **Measurement of outcomes** | **Selection of reported result** | **Overall** |
| --- | --- | --- | --- | --- | --- | --- | --- | --- |
| Tabrizi et al. 2020 | Moderate | Moderate | Low | Moderate | Low | Moderate | Moderate | Moderate |
| Fang et al. 2025 | Serious | Serious | Low | Moderate | Serious | Moderate | Serious | Serious |

Note. ROBINS-I uses Low, Moderate, Serious, Critical, and No information. Moderate is not equivalent to RoB 2 Some concerns, and Serious is not relabelled as RoB 2 High.

**Part C. Source-based rationale for ROBINS-I judgements**

| **Study** | **ROBINS-I domain** | **Judgement** | **Supporting rationale** |
| --- | --- | --- | --- |
| Tabrizi et al. 2020 | Confounding | **Moderate** | Purposive recruitment was reported; assignment to three groups was described as random, but sequence generation and concealment were not reported. Baseline scores and covariance methods reduced but did not eliminate concern. |
| Tabrizi et al. 2020 | Selection of participants | **Moderate** | Eligibility preceded intervention, but purposive recruitment and limited allocation reporting reduced confidence. |
| Tabrizi et al. 2020 | Classification of interventions | **Low** | VR, medication, and no-intervention groups were clearly defined at baseline. |
| Tabrizi et al. 2020 | Deviations from intended interventions | **Moderate** | Participants and personnel were aware of treatment, and adherence and co-interventions were incompletely reported. |
| Tabrizi et al. 2020 | Missing data | **Low** | Immediate post-treatment results were reported for all three groups and no material attrition was described. |
| Tabrizi et al. 2020 | Measurement of outcomes | **Moderate** | A standardized memory test was used, but assessor blinding was not reported. |
| Tabrizi et al. 2020 | Selection of the reported result | **Moderate** | No protocol or prespecified analysis plan was located despite multiple time points and comparisons. |
| Fang et al. 2025 | Confounding | **Serious** | Assignment was not randomized, groups were markedly unequal, and measured baseline comparison did not adequately address residual confounding. |
| Fang et al. 2025 | Selection of participants | **Serious** | Group formation was insufficiently described; 13 records were excluded and observations were balanced after removal without arm-specific reasons. |
| Fang et al. 2025 | Classification of interventions | **Low** | Reward-enhanced VR and conventional VR without reward feedback were clearly defined. |
| Fang et al. 2025 | Deviations from intended interventions | **Moderate** | Participants and instructors were aware of assignment, and adherence or co-intervention information was limited. |
| Fang et al. 2025 | Missing data | **Serious** | Complete-case deletion was used and exclusions were not reported by group or linked to reasons and outcomes. |
| Fang et al. 2025 | Measurement of outcomes | **Moderate** | Standardized computerized tasks and SNAP-IV ratings were used, but assessor or rater blinding was not reported. |
| Fang et al. 2025 | Selection of the reported result | **Serious** | Multiple outcomes, reward combinations, exclusion rules, and analyses were available without a located protocol or prespecified plan. |

Note. Two review authors independently verified the tool-specific assessments, and discrepancies were resolved through discussion and consensus.
